# Nasal pathobiont abundance is a moderate feedlot-dependent indicator of bovine respiratory disease in beef cattle

**DOI:** 10.1186/s42523-025-00387-y

**Published:** 2025-03-15

**Authors:** Ruth Eunice Centeno-Delphia, Natalie Glidden, Erica Long, Audrey Ellis, Sarah Hoffman, Kara Mosier, Noelmi Ulloa, Johnnie Junior Cheng, Josiah Levi Davidson, Suraj Mohan, Mohamed Kamel, Josh I. Szasz, Jon Schoonmaker, Jennifer Koziol, Jacquelyn P. Boerman, Aaron Ault, Mohit S. Verma, Timothy A. Johnson

**Affiliations:** 1 Department of Animal Science, Purdue University, 270 S Russell St, room 2020, West Lafayette, IN USA; 2 Escuela Agrícola Panamericana Zamorano, Valle del Yeguare, Tegucigalpa, Honduras; 3 Department of Agricultural and Biological Engineering, Purdue University, West Lafayette, IN USA; 4 Department of Medicine and Infectious Diseases, Faculty of Veterinary Medicine, Cairo University, Giza, 12211 Egypt; 5 School of Veterinary Medicine, Texas Tech University, Amarillo, TX USA; 6 Five Rivers Cattle Feeding, LLC, Johnstown, CO 80534 USA; 7 Department of Electrical and Computer Engineering, Purdue University, West Lafayette, IN USA; 8 Weldon School of Biomedical Engineering, Purdue University, West Lafayette, IN USA; 9 Brick Nanotechnology Center, Purdue University, West Lafayette, IN USA

**Keywords:** Beef cattle, 16S rRNA gene, qPCR, BRD-pathobionts, Bovine respiratory disease

## Abstract

**Background:**

Bovine respiratory disease (BRD) poses a persistent challenge in the beef cattle industry, impacting both animal health and economic aspects. Several risk factors make an animal susceptible to BRD, including bacteria such as *Mannheimia haemolytica*,* Pasteurella multocida*,* Histophilus somni*, and *Mycoplasma bovis*. Despite efforts to characterize and quantify these bacteria in the nasal cavity for disease diagnosis, more research is needed to understand if there is a pathobiont abundance threshold for clinical signs of respiratory disease, and if the results are similar across feedlots. This study aims to compare the nasal microbiome community diversity and composition, along with the abundance of four bacterial pathogens and associated serotypes, in apparently healthy and BRD-affected beef cattle. Nasal swabs were collected from four beef feedlots across the US, covering the years 2019 to 2022. The study included post-weaned beef cattle with diverse housing conditions.

**Results:**

Quantification of BRD-associated pathogens effectively distinguished BRD-affected from apparently healthy beef cattle, surpassing the efficacy of 16S rRNA gene sequencing of the nasal microbiome community. Specifically, *H. somni*,* M. bovis*, and *M. haemolytica* had higher abundance in the BRD-affected group. Utilizing the abundance of these pathobionts and analyzing their combined abundance with machine learning models resulted in an accuracy of approximately 63% for sample classification into disease status. Moreover, there were no significant differences in nasal microbiome diversity (alpha and beta) between BRD-affected and apparently healthy cattle; instead, differences were detected between feedlots.

**Conclusions:**

Notably, this study sheds light on the beef cattle nasal microbiome community composition, revealing specific differences between BRD-affected and apparently healthy cattle. Pathobiont abundance was increased in some, but not all farms. Nonetheless, more research is needed to determine if these differences are consistent across other studies. Additionally, future research should consider bacterial-viral interactions in the beef nasal metagenome.

**Supplementary Information:**

The online version contains supplementary material available at 10.1186/s42523-025-00387-y.

## Background

Bovine respiratory disease (BRD) can be caused by bacteria, viruses, and fungi, resulting in significant economic losses due to increased morbidity, mortality, treatment costs, and reduced production in beef cattle [1–3]. Specifically in the beef cattle industry, 16.2% of the cattle placed in a feedlot exhibited signs of respiratory disease at some point during the feeding period [3]. In 2013, the average cost of treating respiratory disease for a single case of BRD was $23.60 [3]. Moreover, BRD development involves multifactorial interactions, including predisposing, environmental, and epidemiological factors [2, 4]. Diagnosis of BRD in feedlots typically relies on observing clinical signs and utilizing a scoring method like DART (depression, appetite loss, respiratory character change and rectal temperature) [5–7]. However, the utilization of clinical sign to diagnose BRD has been shown to have a low sensitivity (62%) and specificity (63%) in correctly classifying the BRD status of an animal [8–10]. Thus, the multifactorial development and lack of efficient diagnosis methods make BRD diagnosis, management, and therapy challenging. Studies have suggested that the respiratory microbiome may be an indicator of BRD development or to help diagnose cattle [11–16]. For example, BRD-affected beef cattle have lower nasal alpha diversity compared to apparently healthy cattle [11, 17], potentially indicating that higher microbiome diversity confers resistance to pathogen colonization [18, 19]. Additionally, BRD pathobionts (*H. somni*,* P. multocida*,* M. haemolytica*, and *M. bovis* ) have been associated with BRD cases in the post-mortem lung tissue [11, 20–22]. In some studies, BRD-pathobionts, had higher abundance or prevalence in the upper respiratory tract of cattle diagnosed with BRD than healthy cattle [11, 16]. Thus, it may be possible to determine disease status by measuring pathobiont abundance in easily collectable samples (nasal or nasopharyngeal). Centeno-Martinez et al., (2022) [11] utilized a machine learning approach (Random Forest), using nasal BRD-pathobiont abundance, animal age, and total nasal 16S rRNA gene copies as features to classify each animal as healthy or BRD-affected. The classification sensitivity was 88%, specificity was 55%, and misclassification error was 34%. Additionally, other studies have revealed specific serotypes or strains more often associated with BRD cases. For instance, *M. haemolytica* serotypes A1 and A6 are linked to bovine pneumonic pasteurellosis [23, 24]. *P. multocida*, serotype A3 has been isolated from lung samples collected from BRD mortalities [25, 26] and *H. somni* strain 2336, exhibits pathogenic characteristics [27, 28]. Thus, quantifying these pathobionts and serotypes in upper respiratory samples could enhance BRD detection.

Characterization of the bovine respiratory microbiomes with the use of next-generation sequencing has helped to identify the commensal members present in the respiratory tract. However, the bovine respiratory microbiome could be different, depending on the study or location. As an example, a study characterized the respiratory microbiome of cattle in Canada and determined that *Mycoplasma*, *Lactococcus*,* Moraxella*,* Histophilus*, and *Pasteurella* were the most common genera in the bovine respiratory microbiome samples [29]. Comparatively, a different study in the United States characterized the bovine respiratory microbiome and found that the most common bacterial genera in the bovine respiratory tract were *Mannheimia*,* Mycoplasma*,* Moraxella*,* Psychrobacter*, and *Pseudomonas* [30]. Hence, the aim of this observational study is to characterize the nasal microbiome and assess the abundance of BRD-pathobionts in beef cattle exhibiting clinical signs of BRD, comparing them with healthy animals sampled within the same pen. We hypothesized that if pathobionts reached a certain abundance threshold then respiratory disease would be more common. We made this hypothesis because we assumed that pathobiont abundance would increase due to bacterial proliferation during invasion and disease. Additionally, we hypothesized that animals identified with BRD would have a decrease in nasal microbiome alpha diversity and a shift in beta diversity compared to their healthy pen mates.

## Results

**Fig. 1:**
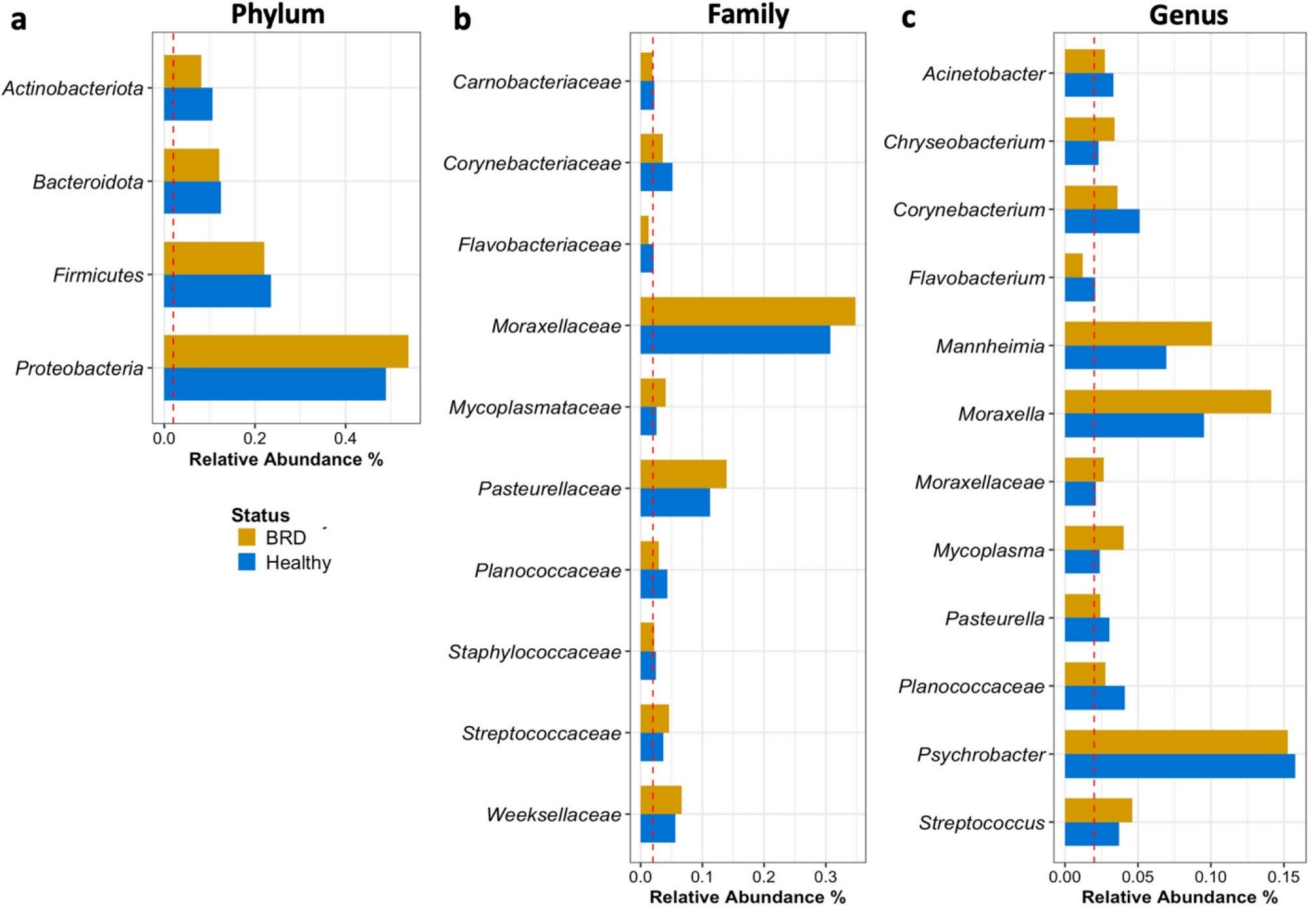
Nasal microbiome taxa with an average relative abundance > 2% per sample at the phylum **(a)**, family **(b)** and genus **(c)** taxonomic levels in both BRD-affected and apparently healthy beef cattle. If only one group (apparently healthy or BRD) surpassed the 2% threshold (red dashed line), then both groups were reported

In this study, the nasal cavity of feedlot cattle was predominantly populated by bacterial members from the *Proteobacteria* phylum, constituting 51% of the community on average, followed by *Firmicutes* (23%), regardless of disease status and feedlot (Fig. 1a). At the family level, *Moraxellaceae* emerged as the most common taxon, comprising 33% of the community, followed by *Pasteurellaceae* (13%), and *Weeksellaceae* (6%). Interestingly, the average relative abundance of these family groups was numerically higher in the BRD-affected group compared to apparently healthy (also referred to as healthy) cattle (Fig. 1b). At the genus level, regardless of disease status and feedlot the group *Psychrobacter* exhibited the highest average relative abundance among all the samples (16%), followed by *Moraxella* (12%) and *Mannheimia* (9%) (Fig. 1c).

**Fig. 2:**
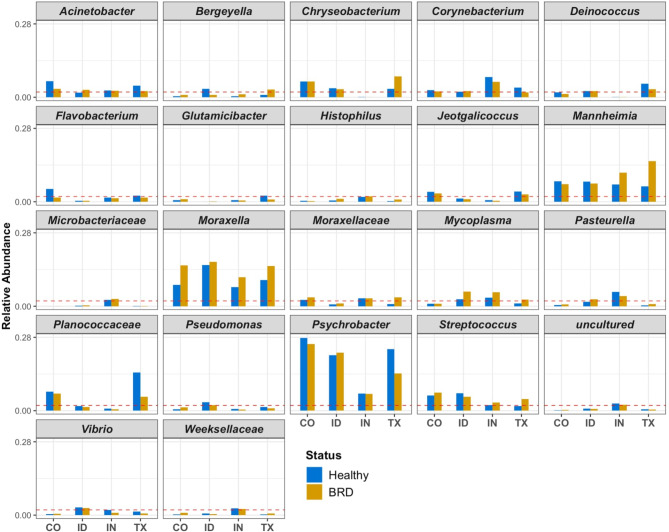
Genera from beef cattle nasal swabs with an average relative abundance > 2% (red dashed line) in at least one health group (BRD-affected and apparently healthy cattle) from at least one of the four feedlots. The relative abundance of all the groups at all feedlots were all plotted to maintain data consistency

Despite the similarities between all the feedlots, the abundance of some genera were different between healthy and BRD animals depending on the feedlot. Principally among these differences was that *Moraxella* relative abundance was numerically higher in all the BRD-affected animals across the four different feedlots, especially CO, IN and TX (Fig. 2). In TX, the relative abundance of *Psychrobacter* and unclassified *Planococcaceae* was much higher numerically in the apparently healthy group (Fig. 2). In the case pathobionts, the relative abundance of *Mannheimia* was numerically higher in the BRD-affected group from IN and TX and the relative abundance of *Mycoplasma* was numerically higher in the BRD-affected cattle from ID, IN, and TX compared to their healthy pen-mates (Fig. 2).

### Relative abundance of BRD-pathobionts present in the beef cattle nasal Microbiome

Samples were separated by feedlot to assess the relative abundance of known BRD-pathobionts, including *Biberstenia*,* Histophilus*,* M. haemolytica*,* M. dispar* (also known as *Mesomycoplasma dispar*), *Mycoplasma*,* M. bovirhinis* (also referred to as *Mycoplasmopsis bovirhinis*), *P. multocida*, and *Trueperella* using 16S rRNA gene taxonomy data (see Additional File 1: Figure S1). One amplicon sequence variant (ASV) was identified as *Mannheimia haemolytica*, and its identity was confirmed as *M. haemolytica* by alignment to the NCBI database (percent identity 99.84%) with the basic local alignment search tool (BLAST). All sequences classified as *Mannheimia* at the genus level were subjected to a multiple sequence alignment to this ASV to determine their similarity to the ASV classified as *M. haemolytica*. A total of 50 ASVs were classified as *Mannheimia*, but only 6 ASVs had > 98% similarity to the ASV identified as *M. haemolytica.* These six ASVs were included to determine the abundance of *M. haemolytica* across different feedlots and disease statuses. Among the four feedlots, IN exhibited the highest relative abundance of pathobionts, while CO had the lowest (see Additional File 1: Figure S1).

### Differentially abundant taxa in the beef cattle nasal cavity

**Fig. 3:**
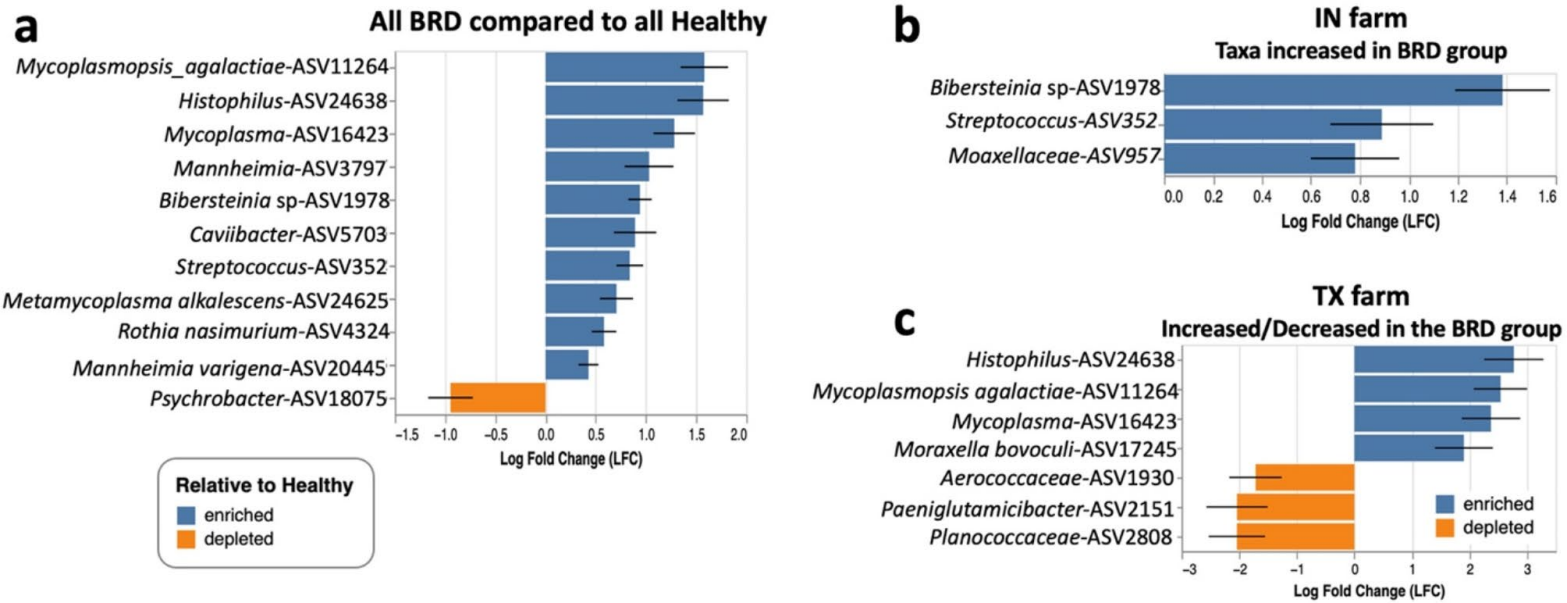
Differentially abundant taxa in BRD-affected relative to apparently healthy cattle when grouping all feedlots (**a**). Differentially abundant in the BRD-affected group compared to the apparently healthy group in IN (**b**) and TX (**c**)

ANCOM-BC analysis was used to identify differentially abundant taxa between BRD-affected and apparently healthy animals in the different feeding facilities. For this analysis, only ASVs with more than 50 counts across all samples were selected. When comparing taxa identified between all the apparently healthy and BRD-affected group in all feedlots, 10 ASVs were significantly increased in the BRD-affected cattle compared to the apparently healthy group (*P <* 0.05). Interestingly, ASVs classified as *Histophilus*,* Mycoplasma*,* Mannheinia* and *Bibersteinia* sp were enriched in the BRD-affected group and one ASV classified as *Psychrobacter* significantly decreased in the BRD-affected group compared to the healthy group (Fig. 3a). After separating the samples by feedlot, BRD-affected cattle from the IN feedlot had a significantly higher abundance of ASVs classified as *Bibersteinia* sp, *Streptococcus* and *Moraxellaceae* (*P <* 0.05) (Fig. 3b). Significant differences were also detected in the TX feedlot, from which, the BRD-affected group had a higher abundance of ASVs classified as *Histophilus*,* Mycoplasmopsis agalactiae*,* Mycoplasma* and *Moraxella bovoculi* (Fig. 3c). No differential abundant taxa were detected in the CO and ID feedlot when considered separately.

### Beef cattle nasal microbiome alpha diversity

In this study, a total of 40,139,159 sequences were detected in DNA extracted from 505 beef nasal swabs collected from CO, ID, IN, and TX. After the denoising step (DADA2) and the removal of *Pseudoalteromonas*, an ASV identified as a contaminant (see Additional File 1: Figure S2), 18 samples were lost (*n* = 487). Of these lost samples, 17 had 0 sequences detected, and 1 was lost during the denoising step. After denoising, a total of 31,619,491 sequences were classified into 25,925 amplicon sequences variants from the remaining samples (*n* = 487). Samples were then rarefied to 10,190 sequences per sample, resulting in a total of 17,622 ASVs that were used to quantify the beef nasal alpha and beta diversity. During the rarefaction step, 19 samples were lost due to low sequence counts (total samples 468, BRD-affected cattle = 225, apparently healthy cattle = 243).

**Fig. 4:**
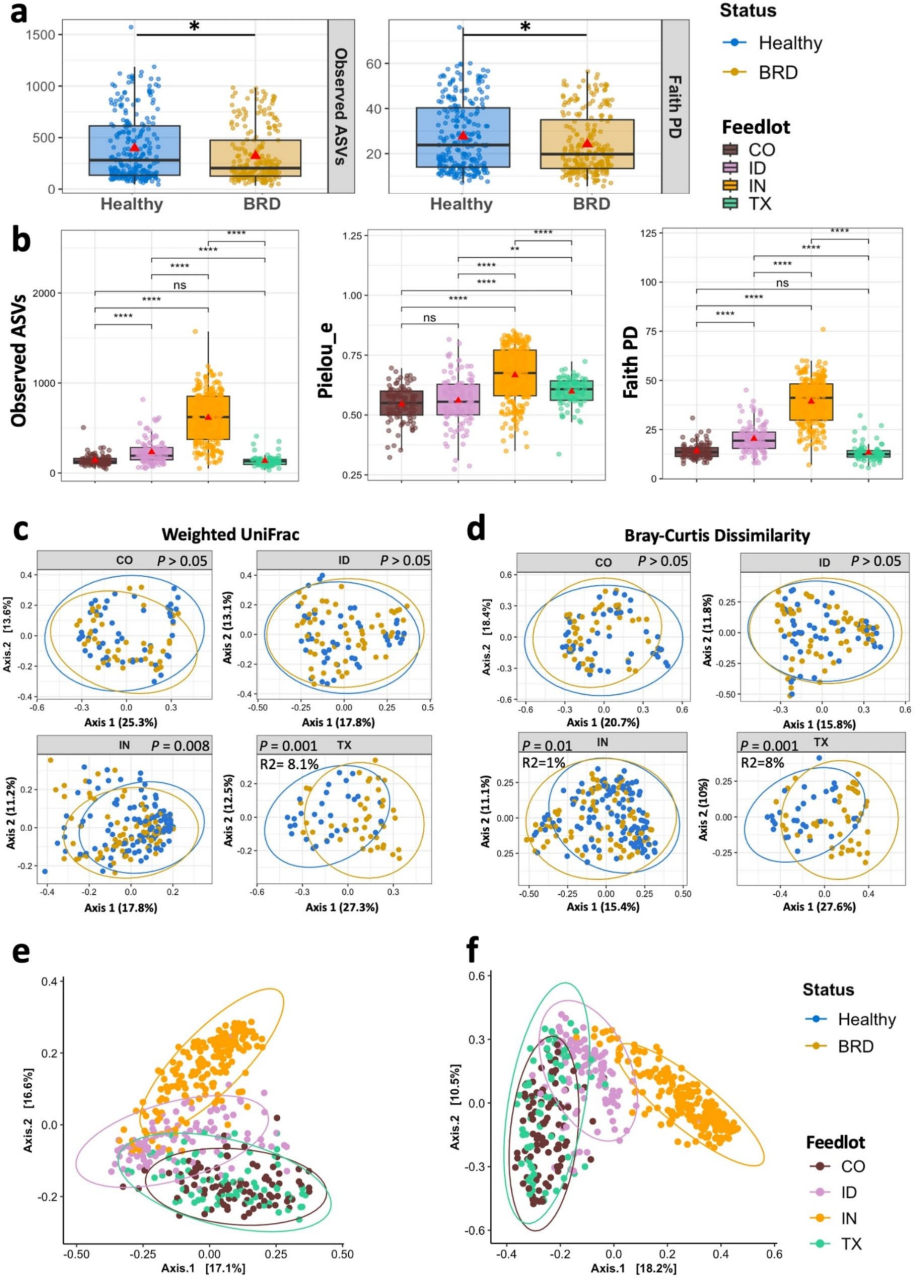
Beef nasal alpha and beta diversity. Alpha diversity between BRD-affected and apparently healthy cattle (**a**) and across all the four feedlots (**b**). Beta diversity between BRD-affected and apparently healthy cattle divided by feedlot and determined by Weighted UniFrac (**c**) and Bray-Curtis Dissimilarity (**d**) and only by feedlot determined by Weighted UniFrac (**e**) and Bray-Curtis Dissimilarity (**f**)

The beef nasal microbiome richness, measured by observed ASVs, and the phylogenetic relationship, measured by Faith PD, in the BRD-affected group was significantly lower than in their apparently healthy pen-mates (Fig. 4a). Based on the group mean for each of the significant alpha diversity results, BRD-affected animals had an 18.8% decrease in Observed ASVs compared to the healthy pen mates. Additionally, the BRD-affected group had a 12.6% mean decrease in their Faith PD value compared to the apparently healthy animals. However, after separating the samples by feedlots and analyzing the effect of disease status on each of the alpha diversity metrics (observed ASVs, Pielou’s evenness measure, which measures community evenness, and Faith PD), no significant differences were detected between the health status groups within each of the feedlots. Samples were separated by feedlot to determine the beef nasal alpha diversity (Fig. 4b). From all four feedlots, IN samples had significantly higher alpha diversity richness, evenness, and phylogenetic diversity compared to all the other feedlots. This group had an observed ASVs mean of 615.12, evenness (Pielou_e) mean of 0.611, and phylogenetic diversity mean of 39.25. Samples collected from TX had the lowest mean values for observed ASVs (135.05), and phylogenetic diversity (13.13) compared to the other feedlots.

### Beef cattle nasal microbiome beta diversity

Nasal bacterial community structure was significantly influenced by disease status, as determined by Weighted UniFrac (F_1,468_ = 3.38, R^2^ = 0.072, *P* = 0.003) and Bray-Curtis dissimilarity (F_1,468_ = 3.54, R^2^ = 0.075, *P* = 0.002) metrics. However, due to the small effect size, no clear separation was observed between the BRD-affected group and the apparently healthy group in the PCoA plots by feedlot (Fig. 4cd). In addition to the effect of disease status, the nasal microbiome structure was different across the feedlots (Weighted UniFrac (F_1,468_ = 39.712, R^2^ = 0.203, *P* = 0.001, Fig. 4e) and Bray-Curtis Dissimilarity (F_1,468_ = 42.356, R^2^ = 0.2146, *P* = 0.001, Fig. 4f)). Interestingly, samples from IN exhibited a distinct community structure compared to CO, ID, and TX. Samples from CO and TX had a similar community structure by both beta diversity metrics and explained by the PCoA plots. Lastly, the dispersion of the samples in each health status group was not significantly different (*P* > 0.05).

### Bacterial BRD-pathobiont prevalence and abundance in the beef nasal cavity determined by qPCR

**Fig. 5:**
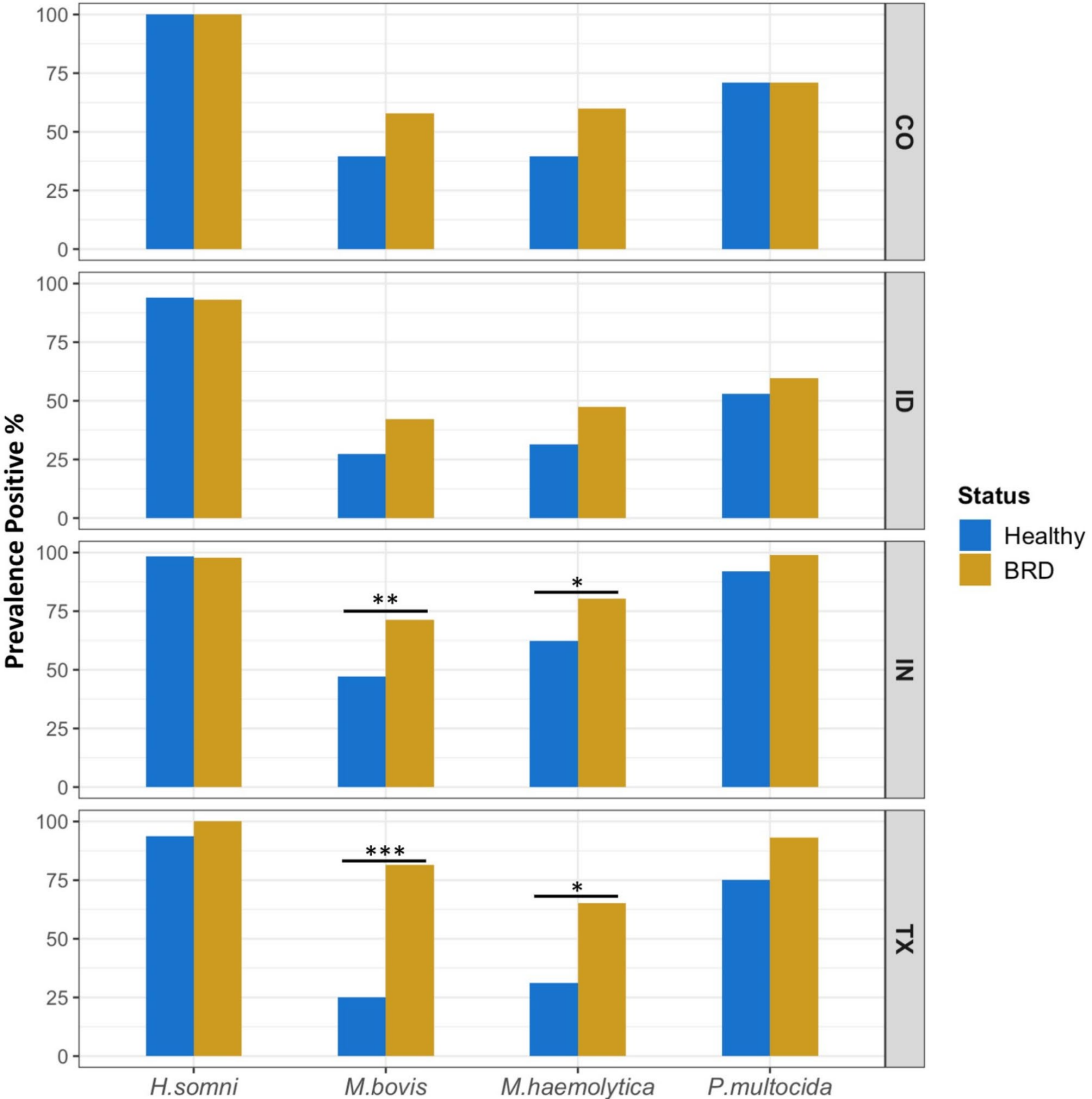
Prevalence of the BRD-pathobionts between apparently healthy and BRD-affected beef cattle sampled in CO, ID, IN, and TX. Prevalence values represent only the cattle that tested positive (present) for each pathobionts. Chi-squared tests were completed to indicate significance: * *P* < 0.05, ** *P* < 0.01, *** *P* < 0.001

The quantification of the four BRD-pathobionts (*H. somni*,* M. bovis*,* M. haemolytica*, and *P. multocida*) was conducted through qPCR using DNA extracted from beef cattle nasal swabs (Fig. 5). Prevalence, indicating presence or absence, was determined using the limit of detection as the cutoff for each individual qPCR assay. In this study, the pathobiont *H. somni* was the most prevalent bacterium regardless of disease status and feedlot, with an overall prevalence of 97.3% (Fig. 5). *P. multocida* was the second most prevalent pathobiont in the cattle nasal cavity, with an average prevalence of 76.68%. The prevalence of *M. bovis* and *M. haemolytica* detected in the IN and in the TX samples was significantly higher (*P <* 0.05) in BRD animals. Although, *P. multocida* seemed numerically greater in the BRD-affected cattle compared to the healthy cattle, there was no statistical difference. No statistical differences were detected for *H. somni. M. bovis* prevalence was higher in the BRD-affected group compared to apparently healthy animals among the four feedlots, with an average prevalence in the BRD-affected group of 63.17% and 34.8% in the apparently healthy cattle. Similar results were observed in *M. haemolytica* prevalence, with BRD-affected animals having a higher mean prevalence (63.17%) than the healthy group (41.15%) (Fig. 5).

**Fig. 6:**
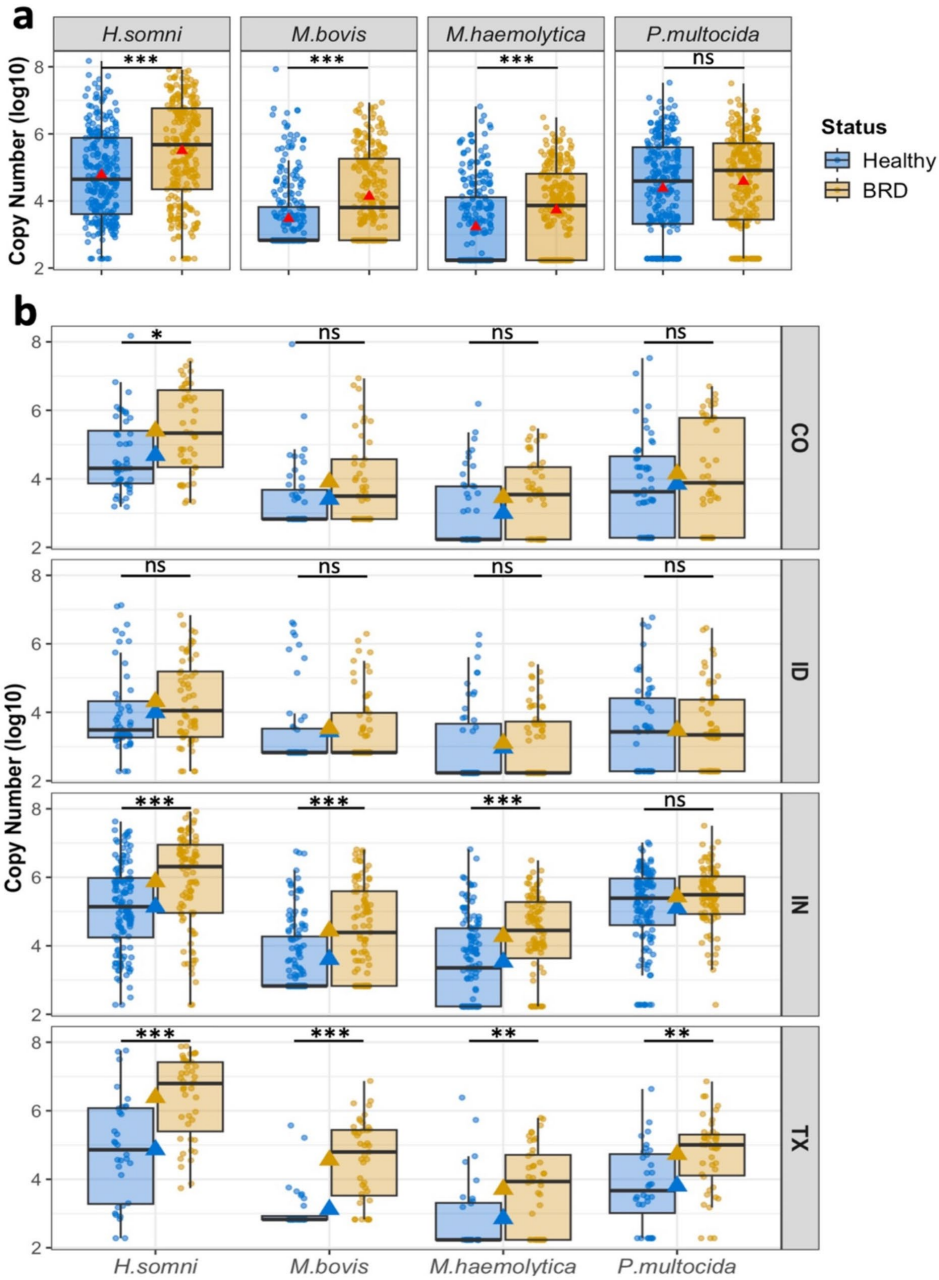
BRD-pathobiont abundance (log_10_) between disease statuses (**a**) and divided by feedlots (**b**). Red, gold, and blue triangles represent the group mean

In this study, the abundance of the pathobionts *H. somni*,* M. bovis*, and *M. haemolytica* was significantly higher in BRD-affected cattle compared to their healthy pen-mates without stratifying the groups by feedlot (Fig. 6a). The mean abundance of *H. somni* was 5.49 log_10_ gene copies in all BRD-affected cattle compared to 4.78 log_10_ gene copies in the healthy group. *M. bovis* mean abundance was 4.13 log_10_ gene copies in the BRD-affected group and 3.47 log_10_ gene copies in the healthy group, and *M. haemolytica* mean abundance was 3.73 log_10_ and 3.22 log_10_ gene copies, respectively. No significant difference in *P. multocida* was detected in the study; both groups had closely similar bacteria carriage, 4.37 log_10_ gene copies in the apparently healthy group and 4.58 log_10_ in the BRD-affected group.

Samples were then separated by feedlot to identify differences in pathobiont abundance between disease statuses within each feedlot (Fig. 6b). Among all the feedlots, BRD-affected animals from TX had a statistically higher (*P* < 0.05) abundance of all four pathobionts (mean abundance range of 3–6 log_10_) compared to their healthy pen-mates (mean abundance range of 2–4 log_10_). Similarly, BRD-affected animals from IN had significantly higher (*P* < 0.05) abundance of *H. somni*,* M. bovis*, and *M. haemolytica* (mean abundance range of 4–6 log_10_), compared to the apparently health animals (mean abundance range 3–5 log_10_) (Fig. 6b). Lastly, the abundance of *H. somni* in CO was significantly higher in the BRD-affected group.

### Prevalence and quantification of *P. multocida* and *M. haemolytica* pathogenic serotypes

Nasal swabs collected from CO (*n* = 82) and ID (*n* = 75) were chosen to quantify the abundance of *P. multocida* serotype A and *M. haemolytica* serotype A1 and A6, aiming to identify differences between BRD-affected and apparently healthy animals, since there were no differences in these feedlots at the species level. Among the three serotypes tested, the prevalence ranged from 100 to 18% (see Additional File 1: Figure S3). The mean abundance of the three serotypes ranged from 2.38 to 4.36 log_10_. (see Additional File 1: Table S1). No statistically significant differences was detected in the abundance of each pathobiont between disease statuses.

### Classification of BRD-affected cattle and apparently healthy using machine learning

In this study, the abundance of BRD-pathobionts was significantly higher in the BRD-affected group compared to their healthy pen-mates. Thus, the abundance of these pathobionts was utilized to create a machine learning classification model using the random forest algorithm to determine the sensitivity, specificity, and classification of using the pathobionts to classify animals as BRD-affected or healthy. A total of ten different tests, each with a unique random seed, were performed. For the random forest, 60% of the samples were randomly selected for the training set, and 40% of the samples were used in the testing set. A total of 31 different features were included in the random forest training data, including the abundance of each of the BRD-pathobionts (copy number and copy log_10_ number), a combination of two, three, and four pathobiont abundances, and their abundance relative to 16S rRNA gene abundance determined by qPCR. Based on the different tests, the mean percent accuracy of the training set was 64.036 ± 2.91 (95% CI: 60.99%, 65.21%). The mean percent accuracy for the testing set was 63.102 ± 2.94 (95% CI: 61.95%, 66.12%). The average sensitivity of the model (correctly identifying BRD animals) was 57.45% ± 5.71%, and the average specificity of the model (correctly identifying healthy animals) was 69.9% ± 6.51%. Additionally, features that were most important to classify the samples as BRD-affected and apparently healthy were identified. From the 31 different features used in the model, the abundance of *H. somni* (copy number log_10_) had the highest effect on the sample classification, followed by the abundance of 16S rRNA gene copy number, *H. somni* copy number, and the combination of *M. bovis* and *M. haemolytica* abundance (Table 1).

**Table 1:** Summary of the most important features in the random forest model based on the mean decrease accuracy (mean value > 1%)

Feature	*n*	Mean	SD	SE	25%*	75%*
HS-copy_log	10	1.37%	0.42%	0.13%	1.06%	1.67%
16 S rRNA copies	10	1.34%	0.45%	0.14%	1.01%	1.66%
HS-copies	10	1.29%	0.34%	0.11%	1.05%	1.53%
HSMH	10	1.20%	0.35%	0.11%	0.95%	1.46%
HSMH_log	10	1.14%	0.36%	0.12%	0.88%	1.40%
MBMH	10	1.12%	0.33%	0.10%	0.88%	1.35%
MBMH_log	10	1.07%	0.18%	0.06%	0.94%	1.19%
HSMB_log	10	1.03%	0.30%	0.10%	0.82%	1.25%
MB-copies	10	1.00%	0.26%	0.08%	0.82%	1.19%

n = number of random forest tests. HS = *H. somni.* MB = *M.bovis.* HSMH = combination of *H.somni* and *M. haemolytica.* HSMH_log = combination of *H.somni* and *M. haemolytica* (copy log_10_). MBMH = combination of *M. bovis* and *M. haemolytica.* MBMH_log = combination of *M. bovis* and *M. haemolytica* (copy log_10_). * 25% and 75% quartiles

## Discussion

BRD poses a significant health concern in the cattle industry, affecting approximately 16.2% of cattle in feedlots [3]. The economic impact of BRD on the industry is estimated to be $800–900 million annually, encompassing expenses related to animal mortality, decreased feed efficiency, and treatment costs [31]. The onset and progression of BRD stems from multiple factors and the presence of bacteria, viruses, and fungi [2, 6, 11, 23, 32–34]. The objective of this study was to characterize the microbiome and the abundance of BRD-pathobionts in the nasal cavity of beef cattle diagnosed with BRD compared to healthy cattle sampled from various beef feedlots in the USA (CO, ID, IN, and TX). We found that BRD-affected cattle exhibited increased abundance of *H. somni*, *M. bovis*, and *M. haemolytica*. When predicting disease status with random forest models, the abundance of these three pathobionts, either alone or summed, contributed to distinguishing animals belonging to each disease status. While nasal microbiome was consistent across feedlots, feedlot location influenced alpha and beta diversity more than disease status. Importantly, the current study provides useful data on the beef cattle respiratory microbiome, as samples were collected from different locations and analyzed using the same pipeline, including sample collection method, DNA extraction, bioinformatics, and statistical analysis.

### Increased abundance of *H. somni*,* M. bovis*, and *M. haemolytica* is associated with BRD cases but dependent on the feedlot

The species *H. somni*,* M. bovis*,* M. haemolytica*, and *P. multocida* have been consistently reported as pathobionts associated with BRD development [6, 21]. Due to their significance in BRD, numerous studies have focused on quantifying their bacterial load in the upper respiratory cavity. These efforts aim to create diagnostic tools for BRD, distinguishing between cattle diagnosed with BRD and their healthy counterparts [11, 12, 15, 16, 35–39]. Most of these studies have been conducted in specific locations worldwide, such as the USA, France, Italy, the United Kingdom, the Netherlands, and Denmark. As previously mentioned, differences in feedlot, including animal handling, diet, environmental factors, housing (individual or in group), temperature when collecting the samples, age or breed could influence nasal microbiome composition; thus, all these factors may be important to identify any changes in the beef nasal microbial community. Therefore, in this study, nasal swabs were collected from different beef feedlots across the USA to determine if the results would be consistent between the feedlots.

Due to the difficulty in sampling the lower respiratory tract, the abundance of BRD-pathobionts in the bovine nasal cavity and nasopharynx have been explored as sampling locations as a proxy for the lower respiratory tract to indicate disease. For example, Goto et al., (2023) [36] found that BRD-affected cattle exhibited more *M. bovis* in nasal swabs than apparently healthy cattle. Pratelli et al., (2021) [16] found that *M. haemolytica* was more prevalent (32.6%) in the BRD animals than in healthy animals (18.3%). Another study performed by Valeris-Chacin et al., (2022) [38] found that the prevalence of *M. bovis* in the nasal cavity collected seven days post-feedlot arrival was associated with a significant increase in *M. haemolytica* prevalence 28 days post-feedlot arrival, indicating a potential pathobiont interaction, or a potential colonization succession, between *M. bovis* and *M. haemolytica*. Interestingly, also in our study, across more than 500 nasal swab samples, it was the prevalence of *M. bovis* and *M. haemolytica* as well as *H. somni* that were higher in BRD-affected animals These findings are consistent with those of Centeno-Martinez et al. (2022) [11], which also reported a higher abundance of *M. bovis* and *M. haemolytica* in BRD-affected cattle.

To further understand if BRD-pathobiont abundance in the nasal cavity can be used to classify BRD-affected and healthy cattle, the abundance of each pathobiont and their interactions (pairwise sums) were included in a random forest model. Our results demonstrated that the abundance of *H. somni* alone or summed with *M. bovis* or *M. haemolytica*, were some of the most important factors to classify beef cattle as either BRD-affected or apparently healthy, with a mean accuracy of 63.15%, mean sensitivity of 57.45, and a mean specificity of 69.9%; similar values were reported in Centeno-Martinez et al. (2022), indicating its potential to improve BRD disease diagnosis [11]. Application of machine learning algorithms to identify BRD cases has been previously tested using animal behavior data, animal weight, sex, information about pen size, or environmental conditions and BRD morbidity risk [40–42]. Thus, the combination of animal behavior data in addition to the abundance of BRD-pathobionts can be used to improve BRD detection. Nonetheless, it is important to mention that the results observed could be applicable to beef cattlestudies; more research is needed to test these results in dairy cattle or in older feedlot cattle.

### Beef nasal taxonomy composition remains stable, with specific members associated with each feedlot and disease status

In this study, the composition of the nasal microbiome in beef cattle across the different feedlots primarily consisted of the phylum *Proteobacteria* and the family group *Moraxellaceae*. At the genus level, *Moraxella*,* Mannheimia*, and *Psychrobacter* were the predominant groups, regardless of disease status. Intriguingly, both *Moraxella* and *Mannheimia* showed higher abundance in animals diagnosed with BRD compared to the apparently healthy cattle, while *Psychrobacter* abundance varied based on the feedlot and disease status. Other studies have determined that *Moraxella*,* Psychrobacter* and *Mannheimia* are considered commensal members of the bovine nasal cavity [11, 43]. Based on our study, ASVs classified as *Mannhemia* and *Moraxella* species were significantly associated with disease status and dependent of the feedlot. The relationship between the presence of *Moraxella* and its connection to BRD is not fully understood; certain studies have suggested a link between this genus and the development of BRD [30, 44, 45]. In particular, one study noted that a high abundance of *Moraxella* in the upper respiratory tract of heifers at feedlot arrival, led to subsequent BRD development [14]. Similarly, Lima et al., (2016) [30] found an association between higher *Moraxella* abundance on day 14 in dairy calves diagnosed with pneumonia, otitis, or pneumonia otitis compared to healthy calves. Additionally, certain *Moraxella* species, including *M. bovoculi* and *Moraxella bovis*, have been identified as the primary etiological agents of infectious bovine keratoconjunctivitis (IBK) [46, 47]. Thus, the association of the *Moraxella* genus with BRD cases and its relationship with other *Moraxella* strains could have potential significance in BRD detection. Based on our study, the relative abundance of the genus *Mannhemia* was numerically elevated in BRD cases, when data from all feedlots was combined (Fig. 1c). However, our investigation reveals that *M. haemolytica*, a recognized BRD-pathobiont [23], did not exhibit the highest abundance among *Mannheimia* species, as its relative abundance was less than 1% per sample. Based on the 16S rRNA gene sequencing data for all samples combined, some *Mannheimia* ASVs (*M. varigena* an unclassified *Mannheimia*) were enriched in the BRD group. The role of *Mannheimia* species, different from *M. haemolytica*, is unclear, and it is possible that they may be commensal members of the nasal microbiome in beef cattle [14]. *Psychrobacter* has been identified in various environments, ranging from different animal microbiomes to seawater, sea ice, marine sediment, glacial ice, and permafrost [48]. Despite its detection in diverse settings, its potential to cause or prevent disease remains unknown [48]. Furthermore, the genus *Psychrobacter* has been recognized as a commensal of the bovine nasal microbiome but is occasionally detected in nasal samples from animals with BRD [11, 43, 49, 50]. However, some studies have even suggested that *Psychrobacter* may have an antagonistic effect on *Mycoplasma* abundance [11, 30, 35]. A separate n vitro study identified that *Psychrobacter* was able to inhibit the growth, motility and biofilm formation of *Pseudomonas aeruginosa* PAO1, a gram negative bacterial pathogen [51]. However, this hypothesis has not been tested in vitro against *Mycoplasma.*

### Feedlot had greater influence on beef nasal alpha and beta diversity than disease status

In addition to identifying any microbiome dysbiosis caused by disease status, changes in alpha and beta diversity have been reported as other indicators of disease development [13, 52, 53]. Based on our results, BRD-affected cattle exhibited a mean decrease of 18.8% in Observed ASVs, and a 12.6% mean decrease in their phylogenetic diversity compared to their healthy pen-mates. Similar findings were reported by Centeno-Martinez et al., (2022) [11], where BRD-affected animals showed a 20% decrease in nasal microbiome richness and an 11% decrease in microbiome phylogenetic diversity. Previous reports have also highlighted the influence of feedlot on nasal microbiome composition [49, 54]. For example, the samples collected for this study originated from different beef feedlots across the USA. Three feedlots (CO, ID, and TX) housed their beef cattle outdoors, while IN housed their cattle indoors. Additionally, samples from IN were collected from 2019 to 2022 (one sample per animal), indicating exposure to diverse environmental conditions, including variations in environmental temperature. Studies have suggested that environmental temperature can affect microbiome composition by influencing bacterial growth and shaping the community [55, 56]. For example, Centeno-Martinez et al., (2022) [11] showed a positive association between average daily temperature and nasal alpha diversity, as well as changes in nasal bacterial structure from samples collected in February to March (transition from Winter to Spring). Conversely, samples from CO were collected in one week in April and in another week in October 2021, ID samples were collected from September to November 2021, and TX samples were collected from March to April 2021. Therefore, incorporating difference in feedlot management and environmental factors is crucial for comprehending changes in the nasal microbiome community.

## Conclusions

Quantifying BRD-pathobionts proved effective in distinguishing between BRD-affected and apparently healthy beef cattle, particularly the IN and TX feedlots. Specifically, the abundance of *H. somni*,* M. bovis*, and *M. haemolytica* was higher in the BRD-affected group compared to the healthy animals, irrespective of the feedlot. When employing the abundance of these pathobionts and their combined abundance to classify samples using machine learning models, the models achieved an accuracy of about 63%, suggesting a potential connection of these microbes in the nasal cavity with BRD diagnosis. Bacterial 16S rRNA gene sequencing of the beef cattle nasal microbiome revealed that community richness and phylogenetic diversity were lower in the BRD-affected group. However, when samples were separated by feedlot, no significant differences were detected, emphasizing that alpha diversity between disease statuses is similar within each feedlot. Similarly, analysis of the nasal bacterial community revealed that feedlot had a more significant influence on the community structure (beta diversity) than disease status, especially with samples from IN exhibiting a dissimilar bacteria community structure compared to samples from CO, ID, and TX. Lastly, samples from this study were collected from different feedlots in the USA and only included beef cattle; thus, it is necessary to conduct similar research incorporating the effect of feedlot including animal management, diet, age, breed or environmental conditions, and both dairy and beef feedlots to determine if the observed patterns in this study remain consistent.

## Materials and methods

### Animal population

Nasal swabs were collected from four different beef feedlots (CO, IN, ID, and TX) in the US from 2019 to 2022, with nearly an equal number of samples from visually healthy animals and BRD-affected animals (Table 2). Each selected animal was sampled only once. Unfortunately, data regarding the animal age were not recorded. Animal selection adhered to the DART (depression, appetite loss, respiratory character, and rectal temperature (> 103 °F)) method outlined in Centeno-Martinez et al., (2022) [11]. One animal was identified with BRD clinical sign, and one or two healthy cattle were sampled from the same pen. All the animals selected for the study were weaned beef cattle. Cattle from IN were housed in covered pens, while cattle from CO, ID, and TX were housed outdoors in pens.

### Cattle nasal swab collection, DNA extraction and bioinformatics analysis

After selecting the animals for the study, the farm personnel collected one double nasal swab from each animal sampled from CO, ID, and TX. For the IN sample, two nasal swabs were collected per animal. Samples were kept in a refrigerator at the farm facilities after collection before being shipped to the lab. They were shipped within 5 days of collection and processed within 7 days. Once the samples arrived at the lab, the samples were stored at 4°C until further processing. Nasal swabs and DNA extraction were processed following the protocol outlined by Centeno-Martinez et al., (2022) [11]. Upon extracting the DNA and obtaining 16S rRNA gene amplicons were sequenced via Illumina MiSeq Sequencer (2 × 250 paired-end) at the Purdue Genomic Core Facility. The V4 variable region of the 16S rRNA gene was amplified for this study. The raw sequences underwent analysis using Quantitative Insight Into Microbial Ecology (QIIME2) v.2022.8. Raw forward and reverse sequences were trimmed and truncated using DADA2 to ensure average sequence quality was > Q30. All sequences were then clustered into Amplicon Sequence Variants (ASVs). Taxonomy was assigned using the 515/806 region of the SILVA 138 database. An assessment of the microbiome composition using bias correction (ANCOM-BC) was conducted to discern variations in abundance among taxa between healthy and BRD-afflicted animals. This approach adjusts for the inherent compositional features of the microbiome community [57, 58]. Prior to this analysis, the ASV table was filtered to remove any ASV with a total sequence count less than 50 (representing 1/100,000 of the dataset). A significance threshold of 0.05 was applied to identify differentially abundant taxa with statistical significance. Calculation of alpha and beta diversity, the beef cattle nasal samples ASV table was rarefied to 10,190 sequences per sample. During this process, some samples were lost due to low sequence count, specifically, 17 samples had 0 sequence counts (total beef samples = 505, after rarefying = 468, Table 2). Alpha diversity was estimated in QIIME2 using Observed features, Faith’s phylogenetic diversity (Faith PD) and Pielou's evenness. Beta diversity was determined using the Bray-Curtis Dissimilarity Index and Weighted UniFrac (incorporating phylogenetic diversity). Beta diversity results were plotted as principal coordinate analysis (PCoA) using R (v. 4.1.2) and RStudio (v. 2021.09.1). The Beta diversity was compared between disease statuses across all the different feedlots. To address the feedlot effect, disease status served as a predictor and feedlot as a within-factor, utilizing the ‘strata’ function. Pairwise comparisons using the ‘pairwise.adonis2’ function were employed as a post-hoc test if the effect of feedlot was significant (*P* ≤ 0.05). A dispersion test was conducted using a permutation test of multivariate homogeneity of group dispersion to detect significant difference in the distance of each sample from the group centroids (BRD or healthy).

**Table 2:** Summary of the total nasal swabs data collection from four different beef feedlots in the US before and after bioinformatics rarefication

Beef feedlot samples before rarefying			Beef feedlot samples after rarefying		
Feedlot	Healthy	BRD	Feedlot	Healthy	BRD
CO (*n* = 93)	48	45	CO (*n* = 93)	48	45
ID (*n* = 108)	51	57	ID (*n* = 105)	49	56
IN (*n* = 218)	125	93	IN (*n* = 197)	115	82
TX (*n* = 86)	40	46	TX (*n* = 73)	31	42

### Analysis of the mock community and negative controls

To ensure PCR amplification and sequencing reliability, a positive control containing 20 known bacterial DNA strains, designated as the mock community (ATCC MSA-1002TM), was included. This mock community was sequenced simultaneously with the beef samples. Raw mock community sequences were analyzed separately to assess amplification and sequence quality. In QIIME2 v.2022.8, we compared the mock sequences with a reference file containing the 16S rRNA gene sequences of the 20 known bacterial strains. Forward and reverse sequences were trimmed to retain those with a quality score > 30. Sequencing quality was evaluated using the ‘evaluate_seqs’ function in QIIME2, aligning observed sequences in the mock samples with the mock reference file to determine matches and mismatches. In addition to the positive control, two types of negative controls were included during DNA extraction and sequencing: empty tubes processed alongside nasal swab samples and PCR water in the PCR amplification step. Empty tubes represented samples processed concurrently with nasal swab samples during DNA extraction, serving as the DNA extraction kit negative control to identify potential contaminants. Both empty tubes and PCR water were sequenced alongside the positive control and beef cattle samples. Raw sequences from empty tubes and PCR water were analyzed separately, following the above procedure, and forward and reverse sequences were trimmed (median quality score < 30) before taxonomy assignment using 515/806 region of the SILVA 138 database. To identify potential contaminants, observed sequences in the mock community and negative controls (empty tubes and PCR water) were utilized, following the methods outlined by Centeno-Martinez et al., (2022) [11]. In this study, one ASV classified as *Pseudoalteromonas* was identified as a contaminant and removed from the samples due to its presence in the DNA extraction negative controls (empty tubes) and beef nasal swabs (see Additional File 1: Figure S2).

### BRD-pathobionts quantification in the beef cattle nasal cavity

The quantification of BRD-pathobionts in the cattle nasal cavity involved constructing a qPCR standard curve using DNA extracted from *P. multocida*,* H. somni*,* M. haemolytica*, and *M. bovis*. The protocol by Centeno-Martinez et al., (2022) [11] was followed to quantify the abundance of the BRD-pathobionts in the beef cattle nasal cavity. Briefly, DNA from pure isolates of *P. multocida*,* H. somni*, and *M. haemolytica* came from the Indiana Animal Disease Diagnostic Laboratory (ADDL) at Purdue University, while DNA from *M. bovis* was obtained from the *M. bovis* strain 25,523 (ATCC). Primers used to target the BRD-pathobionts are listed in Additional File 1: Table S2. For qPCR standard curve construction, PCR assays were performed following the methods in Centeno-Martinez et al., (2022) [11] and the resulting amplicons were cleaned and purified with the Monarch PCR & DNA cleanup kit. A 10-fold serial dilution (10^8^ to 10^0^) was generated for each BRD-pathobiont amplicon to create the qPCR standard curve. Technical replicate assays were performed, and the standard curve was established by linear regression of the average cycle quantification (Cq) of each sample and log_10_ amplicon copies/µl from each dilution. One dilution from each bacterium was used as the positive control, and PCR-grade water served as the negative control in each qPCR triplicate, following the protocol by Centeno-Martinez et al., (2022) [11].

### 16S rRNA gene quantification

The quantification of the bacterial 16S rRNA gene served as a proxy for total bacteria in the cattle nasal cavity. A pool of DNA extracted from various nasal swabs served as the template for the PCR reaction. The 16S rRNA gene PCR was carried out with specific primers (8F and 1492R) targeting the gene and following the protocol by Centeno-Martinez et al., (2022) [11]. For the 16S rRNA gene qPCR standard curve generation, a 10-fold serial dilution of the amplicons were made (10^8^ to 10^0^ copies/µl). The 16S rRNA gene qPCR assays were performed following the protocol by Centeno-Martinez et al., (2022) [11]. Standard curve generation and inclusion of positive and negative controls were consistent with the earlier PCR step. To calculate the abundance of BRD-pathobionts in the nasal cavity, their prevalence was determined based on the cutoff limit of detection (LOD) for each bacterium. Samples with Cq values below the LOD were classified as positive, while those above were deemed negative (see Additional File: Table S3). Additionally, the relative abundance of each BRD-pathobiont was assessed by dividing its copy number by the 16S rRNA gene copy number per sample (BRel) and the sum of the four bacteria copy numbers (PRel). This analysis allowed identification of each bacterium relative abundance in comparison to the total bacterial community (BRel) and relative abundance compared to other BRD-pathobionts (PRel).

### *M. haemolytica* and *P. multocida* pathogenic serotypes quantification

This study aimed to quantify the abundance of BRD-pathobiont pathogenic serotypes (*M. haemolytica* serotypes A1 and A6, and *P. multocida* serotype A) as well as non-pathogenic serotypes (*M. haemolytica* serotypes A2 and A5 and *P. multocida* serotype D). We utilized isolate genome sequences from Sheets (2023) [59] and Wickware (2022) [60] as input to predict serotypes using a server and database created by [61, 62]. We utilized one *P. multocida* isolate predicted as A serotype and another *P. multocida* predicted as serotype D. For *M. haemolytica*, two isolates predicted a serotype A1 and two predicted as A6 were selected, while two different isolates predicted as A2 and A5 serotypes were used as negative controls. Frozen cultures of the *M. haemolytica* and *P. multocida* serotype isolates were streaked on blood-agar medium (Tryptone Soya Agar with sheep blood, Thermofisher Scientific, Waltham, MA, USA) and incubated in a microaerophilic chamber with 5% CO2 at 37 °C for 18 to 24 h. Single colonies were subcultured in liquid culture media 2.8% Brucella Medium Base (Thermofisher Scientific, Waltham, MA, USA) at 37 °C with agitation at 195 rpm. A secondary bacterial culture was performed following the same conditions. Then, DNA was extracted from the bacterial cells following Centeno-Martinez et al., 2022 [11] protocol. To confirm the identity of the isolates, a secondary BRD-pathobiont serotype identification step was performed using the extracted DNA from the pathogenic and non-pathogenic *P. multocida* and *M. haemolytica* serotypes. Briefly, a PCR assays targeting the 16S rRNA gene was amplified using the pathobionts extracted DNA. 16S rRNA gene amplicons (27F and 1492R primers) for each of the pathobionts were sequenced via Sanger sequencing to ensure the isolate was not contaminated. Multiple sequence alignments to known reference sequences were performed for *P. multocida* (*P. multocida* strain P1933 and P030653/1 retrieved from NCBI) and *M. haemolytica* (*M. haemolytica* strain 90826 and 120731, retrieved from NCBI) subspecies identification.

After confirming the identity of each of the isolates, the extracted DNA of *M. haemolytica* serotype A1 (Mh70), and serotype A6 (Mh19) and *P. multocida* serotype A (Pm6) were used to construct a qPCR standard curve to quantify the abundance of each of the pathogenic serotypes. Additionally, DNA was extracted from a non-pathogenic BRD-pathobionts and used as the negative control for PCR and qPCR. A PCR assay was used to generate *P. multocida* serotype A amplicon. A reaction mixture with a total volume of 50 µl was prepared, comprising 32.5 µl of iTaq™ Universal Probes Supermix (BioRad, CA, USA), 12.5 µl of Primer/Probe mix (sourced from Integrated DNA Technologies, IDT), as described in Additional File 1: Table S2, with primer and probe concentrations of 0.5 µmol/µl and 0.3 µmol/µl, respectively [63]. The reaction also included 3 µl of PCR-grade water and 2 µl of nucleic acid template. The PCR cycling parameters followed those outlined by Wang et al. (2023) [63], with an annealing temperature set at 59 °C. Similarly, PCR assay was performed to generate *M. haemolytica* A1 and A6 amplicons. A 50 µl reaction mixture was prepared, consisting of 32.5 µl of LightCycler 480 SYBR Green I Master (Thermo Fisher Scientific, PA, USA), 12.5 µl of both primers at a concentration of 0.8 µM, 3 µl of PCR-grade water, and 2 µl of nucleic acid template. The PCR cycling conditions were conducted according to Klima et al. (2017) [64]. PCR amplicons for each pathobiont were purified using the method outlined by Centeno-Martinez et al. (2022) [11] and diluted serially from 10^8^ to 10^0^. These diluted amplicons were then used to create qPCR standard curves for each pathobiont. The qPCR analysis was conducted in triplicate using a 20 µl reaction volume. For *P. multocida* serotype A, qPCR reaction included 13 µl of iTaq™ Universal Probes Supermix (BioRad, CA, USA). For *M. haemolytica* serotype A1 and A6, LightCycler 480 SYBR Green I Master (Thermo Fisher Scientific, PA, USA) was used. Each reaction included 5 µl of the respective primer/probe combinations (as described in Additional File 1: Table S2) and 2 µl of nucleic acid template. qPCR cycling conditions for each pathogen serotype followed previously established protocols. Primer specificity for BRD-pathobiont serotypes was assessed using samples from CO (*n* = 82) and ID (*n* = 75). The prevalence of each pathogenic serotype was determined using the standard curve LOD as the detection cutoff, following the Additional File 1: Table S4.

### Statistical analysis

Alpha diversity metrics (Observed ASVs, Faith PD, and Pielou) obtained from 16S rRNA gene sequencing and the abundance of BRD-pathobionts were assessed using a Linear Mixed Model [65] using R (v. 4.1.2) and RStudio (v. 2021.09.1). The model incorporated fixed factors such as disease status (BRD or Healthy) and the feedlot (indicating sample collection sites), with a random factor accounting for different intercepts per feedlot. Assumptions of normality and homogeneity of variance were examined using the afex package (v. 1.3-0). When log transformation failed to meet normality assumptions, non-parametric methods were employed. Mann-Whitney tests were used to evaluate the impact of disease status on alpha diversity and BRD-pathobiont/16S rRNA gene abundance. Furthermore, a chi-squared test was conducted to ascertain significant differences in the prevalence of BRD-pathobionts within each feedlot. The analysis was stratified by feedlot (CO, ID, IN, TX) to investigate feedlot-specific differences. Similar tests were applied to assess differences in the abundance of *P. multocida* and *M. haemolytica* pathogenic serotypes between the two groups. To explore the overall differences between feedlots in alpha diversity and abundance, a Kruskal-Wallis test was utilized. P-values were adjusted using the Benjamini-Hochberg method, and statistical significance was defined at *P* ≤ 0.05.

## Electronic supplementary material

Below is the link to the electronic supplementary material.

Supplementary Material 1

## Data Availability

Nasal swabs sequences divided by feedlot (CO, ID, IN and TX) and DNA extraction negative control sequences were deposited in the NCBI sequence read archive (SRA) database as following: CO samples: Bioproject PRJNA1087724, IN samples: Bioproject PRJNA1087741, ID samples: Bioproject PRJNA1087731, and TX samples: Bioproject PRJNA1087752. DNA negative controls are found in the Bioproject PRJNA1075768, Biosamples SAMN40456531- SAMN40456554. Additional files used in data analysis for this study are available at https://github.com/EuniceCenteno/BigBRDProject for reference and reproducibility.

## References

[1] Casella E, Cantor MC, Silvestri S, Renaud DL, Costa JHC. Cost-aware inference of bovine respiratory disease in calves using precision livestock technology. Proc – 18th Annual Int Conf Distrib Comput Sens Syst DCOSS 2022. 2022;109–116. 10.1109/DCOSS54816.2022.00031.

[2] Snowder GD, Van Vleck LD, Cundiff LV, Bennett GL. Bovine respiratory disease in feedlot cattle: environmental, genetic, and economic factors. J Anim Sci. 2006;84:1999–2008. 10.2527/jas.2006-046. 16864858 10.2527/jas.2006-046

[3] USDA. Feedlot 2011. March; 2013. p. 154.

[4] Edward AJ. Respiratory disease in feedlot cattle in the central USA. Bov Pr. 1996;30:5–7.

[5] Ferraro S, Fecteau G, Dubuc J, Francoz D, Rousseau M, Roy JP, Buczinski S. Scoping review on clinical definition of bovine respiratory disease complex and related clinical signs in dairy cows. J Dairy Sci. 2021;104:7095–108. 10.3168/jds.2020-19471. 33741167 10.3168/jds.2020-19471

[6] Griffin D, Chengappa MM, Kuszak J, McVey DS. Bacterial pathogens of the bovine respiratory disease complex. Veterinary Clin North Am - Food Anim Pract. 2010;26:381–94. 10.1016/j.cvfa.2010.04.004. 10.1016/j.cvfa.2010.04.004 20619191

[7] Maier GU, Rowe JD, Lehenbauer TW, Karle BM, Williams DR, Champagne JD, Aly SS. Development of a clinical scoring system for bovine respiratory disease in weaned dairy calves. J Dairy Sci. 2019;102:7329–44. 10.3168/jds.2018-15474. 31202651 10.3168/jds.2018-15474

[8] Loong TW. Understanding sensitivity and specificity with the right side of the brain. BMJ. 2003;327:716–9. 10.1136/bmj.327.7417.716. 14512479 10.1136/bmj.327.7417.716 PMC200804

[9] White BJ, Renter DG. Bayesian Estimation of the performance of using clinical observations and harvest lung lesions for diagnosing bovine respiratory disease in post-weaned beef calves. J Vet Diagn Invest. 2009;21:446–53. 10.1177/104063870902100405. 19564492 10.1177/104063870902100405

[10] Wolfger B, Timsit E, White BJ, Orsel K. A systematic Rreview of bovine respiratory disease diagnosis focused on diagnostic confirmation, early detection, and prediction of unfavorable outcomes in feedlot cattle. Vet Clin North Am - Food Anim Pract. 2015;31:351–65. 10.1016/j.cvfa.2015.05.005. 26210764 10.1016/j.cvfa.2015.05.005

[11] Centeno-Martinez RE, Glidden N, Mohan S, Davidson JL, Fernández-Juricic E, Boerman JP, Schoonmaker J, Pillai D, Koziol J, Ault A, Verma MS, Johnson TA. Identification of bovine respiratory disease through the nasal Microbiome. Anim Microbiome. 2022;4:1–18. 10.1186/s42523-022-00167-y. 35193707 10.1186/s42523-022-00167-y PMC8862248

[12] Cirone F, Padalino B, Tullio D, Capozza P, Surdo M, Lo, Lanave G, Pratelli A. Prevalence of pathogens related to bovine respiratory disease before and after transportation in beef steers: preliminary results. Anim. 2019;9. 10.3390/ANI9121093. 10.3390/ani9121093 PMC6940923 31817737

[13] Holman DB, McAllister TA, Topp E, Wright ADG, Alexander TW. The nasopharyngeal microbiota of feedlot cattle that develop bovine respiratory disease. Vet Microbiol. 2015;180:90–5. 10.1016/j.vetmic.2015.07.031. 26249828 10.1016/j.vetmic.2015.07.031

[14] McMullen C, Alexander TW, Orsel K, Timsit E. Progression of nasopharyngeal and tracheal bacterial microbiotas of feedlot cattle during development of bovine respiratory disease. Vet Microbiol. 2020;248:108826. 10.1016/j.vetmic.2020.108826. 32891954 10.1016/j.vetmic.2020.108826

[15] Pansri P, Katholm J, Krogh KM, Aagaard AK, Schmidt LMB, Kudirkiene E, Larsen LE, Olsen JE. Evaluation of novel multiplex qPCR assays for diagnosis of pathogens associated with the bovine respiratory disease complex. Vet J. 2020;105425. 10.1016/j.tvjl.2020.105425. 256 January 2019:. 10.1016/j.tvjl.2020.105425 PMC7110767 32113583

[16] Pratelli A, Cirone F, Capozza P, Trotta A, Corrente M, Balestrieri A, Buonavoglia C. Bovine respiratory disease in beef calves supported long transport stress: an epidemiological study and strategies for control and prevention. Res Vet Sci. 2021;135:450–5. 10.1016/J.RVSC.2020.11.002. 33203584 10.1016/j.rvsc.2020.11.002

[17] Howe S, Kegley B, Powell J, Chen S, Zhao J. Effect of bovine respiratory disease on the respiratory microbiome: a meta-analysis. Front Cell Infect Microbiol. 2023;13:1223090. 10.3389/FCIMB.2023.1223090/BIBTEX. 37743862 10.3389/fcimb.2023.1223090 PMC10516580

[18] Cardinale BJ, Duffy JE, Gonzalez A, Hooper DU, Perrings C, Venail P, Narwani A, MacE GM, Tilman D, Wardle DA, Kinzig AP, Daily GC, Loreau M, Grace JB, Larigauderie A, Srivastava DS, Naeem S. Biodiversity loss and its impact on humanity. Nature. 2012;486:59–67. 10.1038/nature11148. 22678280 10.1038/nature11148

[19] Knapp S, Winter M, Klotz S. Increasing species richness but decreasing phylogenetic richness and divergence over a 320-year period of urbanization. J Appl Ecol. 2017;54:1152–60. 10.1111/1365-2664.12826.

[20] Gaudino M, Nagamine B, Ducatez MF, Meyer G. Understanding the mechanisms of viral and bacterial coinfections in bovine respiratory disease: a comprehensive literature review of experimental evidence. Vet Res 2022 531. 2022;53:1–25. 10.1186/s13567-022-01086-1. 10.1186/s13567-022-01086-1 PMC9449274 36068558

[21] Klima CL, Zaheer R, Cook SR, Booker CW, Hendrick S, Alexander TW, McAllister TA. Pathogens of bovine respiratory disease in North American feedlots conferring multidrug resistance via integrative conjugative elements. J Clin Microbiol. 2014;52:438–48. 10.1128/JCM.02485-13. 24478472 10.1128/JCM.02485-13 PMC3911356

[22] Mosier D. Review of BRD pathogenesis: the old and the new. Anim Heal Res Rev. 2014;24:166–9. 10.1017/S1466252314000176. 10.1017/S1466252314000176 25351390

[23] Rice JA, Carrasco-Medina L, Hodgins DC, Shewen PE. *Mannheimia haemolytica* and bovine respiratory disease. Anim Health Res Reviews. 2008;8:117–28. 10.1017/S1466252307001375. 10.1017/S1466252307001375 18218156

[24] Zecchinon L, Fett T, Desmecht D. How *Mannheimia haemolytica* defeats host defence through a kiss of death mechanism. Vet Res. 2005;36:133–56. 10.1051/vetres:2004065. 15720968 10.1051/vetres:2004065

[25] Dabo SM, Taylor JD, Confer AW. *Pasteurella multocida* and bovine respiratory disease. Anim Heal Res Rev. 2008;8:129–50. 10.1017/S1466252307001399. 10.1017/S1466252307001399 18218157

[26] Kumar AA, Shivachandra SB, Biswas A, Singh VP, Singh VP, Srivastava SK. Prevalent serotypes of *Pasteurella multocida* isolated from different animal and avian species in India. Vet Res Commun. 2004;28:657–67. 10.1023/B:VERC.0000045959.36513.e9. 15609866 10.1023/b:verc.0000045959.36513.e9

[27] Siddaramappa S, Challacombe JF, Duncan AJ, Gillaspy AF, Carson M, Gipson J, Orvis J, Zaitshik J, Barnes G, Bruce D, Chertkov O, Detter JC, Han CS, Tapia R, Thompson LS, Dyer DW, Inzana TJ. Horizontal gene transfer in *Histophilus somni* and its role in the evolution of pathogenic strain 2336, as determined by comparative genomic analyses. BMC Genomics. 2011;12:1–20. 10.1186/1471-2164-12-570. 10.1186/1471-2164-12-570 PMC3339403 22111657

[28] Zekarias B, Mattoo S, Worby C, Lehmann J, Rosenbusch RF, Corbeil LB. *Histophilus somni* IbpA DR2/Fic in virulence and Immunoprotection at the natural host alveolar epithelial barrier. Infect Immun. 2010;78:1850–8. 10.1128/IAI.01277-09. 20176790 10.1128/IAI.01277-09 PMC2863524

[29] McMullen C, Orsel K, Alexander TW, van der Meer F, Plastow G, Timsit E. Evolution of the nasopharyngeal bacterial microbiota of beef calves from spring processing to 40 days after feedlot arrival. Vet Microbiol. 2018;225:139–48. 10.1016/j.vetmic.2018.09.019. 30322526 10.1016/j.vetmic.2018.09.019

[30] Lima SF, Teixeira AGV, Higgins CH, Lima FS, Bicalho RC. The upper respiratory tract Microbiome and its potential role in bovine respiratory disease and otitis media. Sci Rep. 2016;6:1–12. 10.1038/srep29050. 27363739 10.1038/srep29050 PMC4929571

[31] Chirase NK, Greene LW. Influence of oral natural interferon-alpha on performance and rectal temperature of newly received beef steers. American Society of Animal Science, Western Section;; 2000.

[32] Bowland SL, Shewen PE. Bovine respiratory disease: commercial vaccines currently available in Canada. Can Vet J. 2000;41:33–48. 10642871 PMC1476343

[33] Centeno-Martinez RE, Mohan S, Davidson JL, Schoonmaker JP, Ault A, Verma MS, Johnson TA. The bovine nasal fungal community and associations with bovine respiratory disease. Front Vet Sci. 2023;10:1–13. 10.3389/fvets.2023.1165994. 10.3389/fvets.2023.1165994 PMC10335396 37441557

[34] Kano R, Konishi K, Nakata K, Sano K, Komatsu S, Nomura M, Okuzumi K, Hasegawa A. Isolation of *Candida krusei* from a case of bovine Bronchopneumonia in a one-year-old heifer. Vet Rec. 2001;148:636. 10.1136/vr.148.20.636. 11394804 10.1136/vr.148.20.636

[35] Centeno-Martinez RE, Klopp RN, Koziol J, Boerman JP, Johnson TA. Dynamics of the nasopharyngeal Microbiome of apparently healthy calves and those with clinical symptoms of bovine respiratory disease from disease diagnosis to recovery. Front Vet Sci. 2023;10:1–12. 10.3389/fvets.2023.1297158. 10.3389/fvets.2023.1297158 PMC10687565 38033643

[36] Goto Y, Fukunari K, Suzuki T. Multiplex RT-qPCR application in early detection of bovine respiratory disease in healthy calves. Viruses. 2023;15:669. 10.3390/v15030669. 36992378 10.3390/v15030669 PMC10057971

[37] Thomas AC, Bailey M, Lee MRF, Mead A, Morales-Aza B, Reynolds R, Vipond B, Finn A, Eisler MC. Insights into *Pasteurellaceae* carriage dynamics in the nasal passages of healthy beef calves. Sci Rep. 2019;9:1–14. 10.1038/s41598-019-48007-5. 31420565 10.1038/s41598-019-48007-5 PMC6697682

[38] Valeris-Chacin R, Powledge S, McAtee T, Morley PS, Richeson J. *Mycoplasma bovis* is associated with *Mannheimia haemolytica* during acute bovine respiratory disease in feedlot cattle. Front Microbiol. 2022;13:946792. 10.3389/FMICB.2022.946792. 35979489 10.3389/fmicb.2022.946792 PMC9376970

[39] Wisselink HJ, Cornelissen JBWJ, van der Wal FJ, Kooi EA, Koene MG, Bossers A, Smid B, de Bree FM, Antonis AFG. Evaluation of a multiplex real-time PCR for detection of four bacterial agents commonly associated with bovine respiratory disease in Bronchoalveolar lavage fluid. BMC Vet Res. 2017;13. 10.1186/s12917-017-1141-1. 10.1186/s12917-017-1141-1 PMC5508684 28705198

[40] Cantor M, Casella E, Silvestri S, Renaud DL, Costa JHC. Using machine learning and behavioral patterns observed by automated feeders and accelerometers for the early indication of clinical bovine respiratory disease status in preweaned dairy calves. Front Anim Sci. 2022;3:852359. 10.3389/fanim.2022.852359.

[41] Cantor M, Costa J. Daily behavioral measures recorded by precision technology devices May indicate bovine respiratory disease status in preweaned dairy calves. J Dairy Sci. 2022;105:6070–82. 10.3168/jds.2021-20798. 35282905 10.3168/jds.2021-20798

[42] Rojas HA, White BJ, Amrine DE, Larson RL. Predicting bovine respiratory disease risk in feedlot cattle in the first 45 days post arrival. Pathogens. 2022;11. 10.3390/PATHOGENS11040442. 10.3390/pathogens11040442 PMC9029152 35456116

[43] McMullen C, Alexander TW, Léguillette R, Workentine M, Timsit E. Topography of the respiratory tract bacterial microbiota in cattle. Microbiome. 2020;8. 10.1186/s40168-020-00869-y. 10.1186/s40168-020-00869-y PMC7288481 32522285

[44] Mcdaneld TG, Kuehn LA, Keele JW. Evaluating the Microbiome of two sampling locations in the nasal cavity of cattle with bovine respiratory disease complex (BRDC) 1. J Anim Sci. 2018;96:1281–7. 10.1093/JAS/SKY032. 29659872 10.1093/jas/sky032 PMC6140963

[45] Zeineldin M, Lowe J, De Godoy M, Maradiaga N, Ramirez C, Ghanem M, Abd El-Raof Y, Aldridge B. Disparity in the nasopharyngeal microbiota between healthy cattle on feed, at entry processing and with respiratory disease. Vet Microbiol. 2017;208:30–7. 10.1016/j.vetmic.2017.07.006. 28888646 10.1016/j.vetmic.2017.07.006

[46] Angelos JA. *Moraxella bovoculi* and infectious bovine keratoconjunctivitis: cause or coincidence? Vet clin North Am -. Food Anim Pract. 2010;26:73–https. 10.1016/j.cvfa.2009.10.002. 10.1016/j.cvfa.2009.10.002 20117543

[47] Cintia Postma G, Ce´sar J, Carfagnini C, Minatel L. *Moraxella bovis* pathogenicity: an update. Comp Immunol Microbiol Infect Dis. 2008;31:449–58. 10.1016/j.cimid.2008.04.001. 18514312 10.1016/j.cimid.2008.04.001

[48] Welter DK, Ruaud A, Henseler ZM, Jong HN, De, Groot P, van Michaux C, Gormezano J, Waters L, Youngblut JL, Ley ND. Free-living, psychrotrophic bacteria of the genus *Psychrobacter* are descendants of pathobionts. mSystems. 2021;6:2. 10.1128/MSYSTEMS.00258-21. 10.1128/mSystems.00258-21 PMC8546975 33850039

[49] Chai J, Liu X, Usdrowski H, Deng F, Li Y, Zhao J. Geography, niches, and transportation influence bovine respiratory Microbiome and health. Front Cell Infect Microbiol. 2022;12:961644. 10.3389/FCIMB.2022.961644. 36171758 10.3389/fcimb.2022.961644 PMC9510686

[50] Nicola I, Cerutti F, Grego E, Bertone I, Gianella P, D’Angelo A, Peletto S, Bellino C. Characterization of the upper and lower respiratory tract microbiota in Piedmontese calves. Microbiome. 2017;5:152. 10.1186/S40168-017-0372-5. 29157308 10.1186/s40168-017-0372-5 PMC5697440

[51] Packiavathy IASV, Kannappan A, Thiyagarajan S, Srinivasan R, Jeyapragash D, Paul JBJ, Velmurugan P, Ravi AV. AHL-lactonase producing *Psychrobacte*r Sp. from Palk Bay sediment mitigates quorum Sensing-mediated virulence production in gram negative bacterial pathogens. Front Microbiol. 2021;12:634593. 10.3389/fmicb.2021.634593. 33935995 10.3389/fmicb.2021.634593 PMC8079732

[52] McMullen C, Orsel K, Alexander TW, van der Meer F, Plastow G, Timsit E. Comparison of the nasopharyngeal bacterial microbiota of beef calves Raised without the use of antimicrobials between healthy calves and those diagnosed with bovine respiratory disease. Vet Microbiol. 2019;231:56–62. 10.1016/j.vetmic.2019.02.030. 30955824 10.1016/j.vetmic.2019.02.030

[53] Timsit E, Workentine M, van der Meer F, Alexander T. Distinct bacterial metacommunities inhabit the upper and lower respiratory tracts of healthy feedlot cattle and those diagnosed with Bronchopneumonia. Vet Microbiol. 2018;221:105–13. 10.1016/j.vetmic.2018.06.007. 29981695 10.1016/j.vetmic.2018.06.007

[54] Gupta VK, Paul S, Dutta C. Geography, ethnicity or subsistence-specific variations in human Microbiome composition and diversity. Front Microbiol. 2017;8:1162. 10.3389/fmicb.2017.01162. 28690602 10.3389/fmicb.2017.01162 PMC5481955

[55] Dickson RP, Erb-Downward JR, Prescott HC, Martinez FJ, Curtis JL, Lama VN, Huffnagle GB. Analysis of culture-dependent versus culture-independent techniques for tdentification of bacteria in clinically obtained bronchoalveolar lavage fluid. J Clin Microbiol. 2014;52(10), 2020. 10.1128/JCM.01028-14 10.1128/JCM.01028-14 PMC4187760 25078910

[56] Proctor DM, Relman DA. The landscape ecology and microbiota of the human nose, mouth, and throat. Cell Host Microbe. 2017;21:421–32. 10.1016/J.CHOM.2017.03.011. 28407480 10.1016/j.chom.2017.03.011 PMC5538306

[57] Mandal S, Van Treuren W, White RA, Eggesbø M, Knight R, Peddada SD. Analysis of composition of microbiomes: a novel method for studying microbial composition. Microb Ecol Health Dis. 2015;26. 10.3402/MEHD.V26.27663. 10.3402/mehd.v26.27663 PMC4450248 26028277

[58] Lin H, Peddada S, Das. Analysis of compositions of microbiomes with bias correction. Nat Commun. 2020;11:1–11. 10.1038/s41467-020-17041-7. 32665548 10.1038/s41467-020-17041-7 PMC7360769

[59] Sheets TR. Leveraging of machine learning to evaluate genotypic-phenotypic concordance of *Pasteurella multocida* isolated from bovine respiratory disease cases. Purdue Univeristy Grad Sch. 2023. https://www.proquest.com/docview/2838439262/previewPDF/C6F93E1FF9734B04PQ/1?accountid=13360%26sourcetype=Dissertations & Theses. Accessed 13 Dec 2023. 10.1016/j.vetmic.2026.111136 42470920

[60] Wickware CL. Applied bacterial ecology in livestock system. Purdue Univeristy Grad Sch. 2022. 10.25394/PGS.21402009.V1. Thesis.

[61] Christensen H, Bisgaard M, Menke T, Liman M, Timsit E, Foster G, Olsen JE. Prediction of *Mannheimia haemolytica* serotypes based on whole genomic sequences. Vet Microbiol. 2021;262:109232. 10.1016/j.vetmic.2021.109232. 34509701 10.1016/j.vetmic.2021.109232

[62] Christensen H, Sajid SM, Bisgaard M, Magistrali CF, Massacci FR, Liman M, Menke T, Bischoff H, Olsen JE. Prediction of *Pasteurella multocida* serotypes based on whole genomic sequences. Vet Microbiol. 2022;271. 10.1016/J.VETMIC.2022.109492. 10.1016/j.vetmic.2022.109492 35714528

[63] Wang H, Xin L, Wu Y, Liu Y, Yao W, Zhang H, Hu Y, Tong R, Zhu L. Construction of a one-step multiplex real-time PCR assay for the detection of serogroups A, B, and E of *Pasteurella multocida* associated with bovine pasteurellosis. Front Vet Sci. 2023;10. 10.3389/fvets.2023.1193162. 10.3389/fvets.2023.1193162 PMC10336434 37448584

[64] Klima CL, Zaheer R, Briggs RE, McAllister TA. A multiplex PCR assay for molecular capsular serotyping of *Mannheimia haemolytica* serotypes 1, 2, and 6. J Microbiol Methods. 2017;139:155–60. 10.1016/j.mimet.2017.05.010. 28551457 10.1016/j.mimet.2017.05.010

[65] Singmann H, Bolker B, Westfall J, Aust F, Ben-Shachar MS, Højsgaardm S, Fox J, Lawrence MA, Mertens U, Love J, Lenth R, Bojesen Christensen RH. Package afex Title Analysis of Factorial Experiments, 2021.

