# Supplementary material for: Nasal pathobiont abundance is a moderate feedlot-dependent indicator of bovine respiratory disease in beef cattle
Source: Anim Microbiome. 2025 Mar 15;7:27. doi: 10.1186/s42523-025-00387-y (PMC11909826; doi:10.1186/s42523-025-00387-y)
Supplement: Supplementary file 1 — Supplementary Material 1 [file 42523_2025_387_MOESM1_ESM.docx]

**ADDITIONAL FILE 1**

**Nasal Pathobiont Abundance is a Moderate Feedlot-dependent Indicator of Bovine Respiratory Disease in Beef Cattle**

Ruth Eunice Centeno-Delphia^1^, Natalie Glidden^1^, Erica Long^1^, Audrey Ellis^1^, Sarah Hoffman^1^, Kara Mosier^1^, Noelmi Ulloa^2^, Johnnie Junior Cheng^1^ Josiah Levi Davidson^3^, Suraj Mohan^3^, Mohammed Kamel^3^, Jon Schoonmaker^1^, Jennifer Koziol^4^, Aaron Ault^6^, Jacquelyn P Boerman^1^, Mohit S Verma^3,5,7^, Timothy A Johnson^1*^.

**Table S1.** Abundance (log_10_) of the *M. haemolytica* and *P. multocida* pathogenic serotypes in the beef nasal cavity samples in CO and ID.

**Table S2.** Primers used to quantify the abundance of the BRD-pathobionts and *M. haemolytica* and *P. multocida* serotypes.

**Table S3.** Limit of detection for each of the BRD-pathobionts and total bacteria abundance in the cattle nasal cavity quantified by qPCR.

**Table S4.** Quantification of *P. multocida* serotype A and *M. haemolytica* serotype A1 and A6 via qPCR.

**Figure S1.** Relative abundance of BRD-pathobionts predicted by 16S rRNA gene sequencing in the beef cattle nasal microbiome.

**Figure S2.** Bar plot showing relative abundance of the ASVs found in the controls including *Pseudoalteromonas*. Controls included the water samples “water”, mock, and empty swabs (Beef and extra).

**Figure S3.** Relative abundance of *M. bovis* **(a)** and *P. multocida* **(b)** based on the 16S rRNA abundance *and M. haemolytica* relative abundance based on the four pathobionts **(c).**

**Figure S4.** Relative abundance of *H.somni* and *P. multocida* based on the four pathobionts from the samples collected in IN **(a,c)** and TX **(b,d).**

**Figure S5.** Total bacteria abundance quantified by 16S rRNA qPCR among the four farms **(a)** and between both disease statuses in IN **(b).**

**Figure S6**. Prevalence of *M. haemolytica* serotype A1 and A6 and *P. multocida* serotype A in samples collected from beef cattle in CO and ID.

**Table S1.** Abundance (log_10_) of the *M. haemolytica* and *P. multocida* pathogenic serotypes in the beef nasal cavity samples in CO and ID.

| **Farm** | **Serotype** | **Status** | **n** | **Mean** | **SD** | **SE** | **25%*** | **75%*** |
| --- | --- | --- | --- | --- | --- | --- | --- | --- |
| CO | Mh-A1 | Healthy | 37 | 2.657 | 1.055 | 0.173 | 2.306 | 3.009 |
|  |  | BRD | 45 | 2.828 | 1.114 | 0.166 | 2.493 | 3.163 |
|  | Mh-A6 | Healthy | 37 | 3.313 | 1.190 | 0.196 | 2.916 | 3.709 |
|  |  | BRD | 45 | 3.186 | 0.912 | 0.136 | 2.912 | 3.460 |
|  | Pm-A | Healthy | 37 | 4.172 | 0.924 | 0.152 | 3.864 | 4.480 |
|  |  | BRD | 45 | 4.363 | 1.069 | 0.159 | 4.042 | 4.684 |
| ID | Mh-A1 | Healthy | 38 | 2.380 | 0.553 | 0.090 | 2.198 | 2.562 |
|  |  | BRD | 37 | 2.545 | 0.558 | 0.092 | 2.359 | 2.731 |
|  | Mh-A6 | Healthy | 38 | 2.753 | 0.895 | 0.145 | 2.459 | 3.048 |
|  |  | BRD | 37 | 2.721 | 0.661 | 0.109 | 2.501 | 2.941 |
|  | Pm-A | Healthy | 38 | 3.974 | 1.226 | 0.199 | 3.571 | 4.377 |
|  |  | BRD | 37 | 3.832 | 1.057 | 0.174 | 3.480 | 4.185 |

*25% and 75% quartile

**Table S2.** Primers used to quantify the abundance of the BRD-pathobionts and *M. haemolyticai* and *P. multocida* serotypes.

| **Target** | **Target** | **Primer name** | **Sequence (5’-3’)** | **Size/legnth** | **Ref** |
| --- | --- | --- | --- | --- | --- |
|  | **gene** |  |  | **(bp)** |  |
| *M. haemolytica* | *sod*A | Mh-SGF | ﻿AGCAGCGACTACTCGTGTTGGTTCAG | 26 | 1 |
|  |  | Mh-SGR | *﻿*AAGACTAAAATCGGATAGCCTGAAACGCCTG | 31 |  |
|  |  | ﻿Mh-BV1P^Ψ^ | *﻿*TTCAACCGCTAACCAGGACAACCCAC | 26 |  |
| *P. multocida* | 16S rRNA | Pm-TMF | ﻿CGCAGGCAATGAATTCTCTTC | 21 | 2 |
|  |  | Pm-TMR | ﻿GGCGCTCTTCAGCTGTTTTT | 20 |  |
|  |  | ﻿Pm-TMP^Ψ^ | ﻿ACTGCACCAACAAATGCTTGCTGAGTTAGC | 30 |  |
| *H. somni* | 16S rRNA | Hs-TMF | ﻿AGGAAGGCGATTAGTTTAAGAGATTAATT | 29 | 2 |
|  |  | Hs-TMR | ﻿TCACACCTCACTTAAGTCACCACCT | 25 |  |
|  |  | ﻿Hs-TMP^Ψ^ | ﻿ATTGACGATAATCACAGAAGAAGCACCGGC | 30 |  |
| *M. bovis* | *opp*D | ﻿PMB996-F | ﻿TCAAGGAACCCCACCAGAT | 19 | 3 |
|  |  | ﻿PMB1066-R | ﻿AGGCAAAGTCATTTCTAGGTGCAA | 24 |  |
|  |  | ﻿Mbovis1016^Ψ^ | ﻿TGGCAAACTTACCTATCGGTGACCCT | 26 |  |
| *M. haemolytica* A1 | *HyP* | HyP_F | ﻿﻿CATTTCCTTAGGTTCAGC | 306 | 4 |
|  |  | HyP_R | ﻿﻿CAAGTCATCGTAATGCCT |  |  |
| *M. haemolytica* A6 | *TupA* | ﻿TupA_F | *﻿*﻿TGAGAATTTCGACAGCACT | 78 | 4 |
|  |  | TupA_R | ﻿﻿ACCTTGGCATATCGTACC |  |  |
| *P. multocida* A | HyaD | HyaD_F | ﻿CAGTTTCTCTGGATTGGCGC | 100 | 5 |
|  |  | HyaD_R | ﻿AAAGCAACATTACCCGCCG |  |  |
|  |  | HyaD-Probe* | ﻿FAM-CTCCGCTTATCCGATTCGCCTTTCC-BHQ1 |  |  |
| ^1^Guenther et al., 2008, ^2^ Mahony et al., 2007, ^3^ Sachse et al., 2010, ^4^ Klima et al., 2017, *^5^* Wang et al., 2023 | | | | | |
| ﻿^Ψ^ Probe fluorophore and double quencher: 5′ 6-FAM/ZEN/3′ IBFQ. * Probe fluorophore | | | | | |

**Table S3.** Limit of detection for each of the BRD-pathobionts and total bacteria abundance in the cattle nasal cavity quantified by qPCR.

| **qPCR assay** |  | ***P. multocida*** | ***H. somni*** | ***M. haemolytica*** | ***M. bovis*** | **16S rRNA gene** |
| --- | --- | --- | --- | --- | --- | --- |
| Standard equation | Slope | -0.313 | -0.270 | -0.316 | -0.297 | -0.324 |
|  | Intercept | 11.784 | 11.182 | 11.38 | 11.172 | 11.699 |
|  | Replicates | 3 | 3 | 3 | 3 | 3 |
| Efficiency (%) |  | 107.28 | 86.74 | 107.96 | 98.40 | 113.11 |
| Dilutions |  | 9 | 9 | 9 | 9 | 9 |
| LOD^Ψ^ |  | 32.863 | 37.385 | 31.76 | 32.51 | 30.52 |
| ^Ψ^ Limit of Detection, Last Cq value in Standard Curve (10^0^) | | | | | | |

**Table S4.** Quantification of *P. multocida* serotype A and *M. haemolytica* serotype A1 and A6 via qPCR.

| **qPCR assay** |  | ***P. multocida* A** | ***M. haemolytica* A1** | ***M. haemolytica.* A6** |
| --- | --- | --- | --- | --- |
| Standard equation | Slope | -0.308 | -0.289 | -0.296 |
|  | Intercept | 11.767 | 10.2698 | 10.588 |
|  | Replicates | 3 | 3 | 3 |
| Efficiency (%) |  | 103.350 | 94.93 | 98.008 |
| Dilutions |  | 11 | 11 | 11 |
| LOD^Ψ^ |  | 36.15 | 34.756 | 34.59 |

^Ψ^: Limit of Detection, Last Cq value in Standard Curve (10^0^)


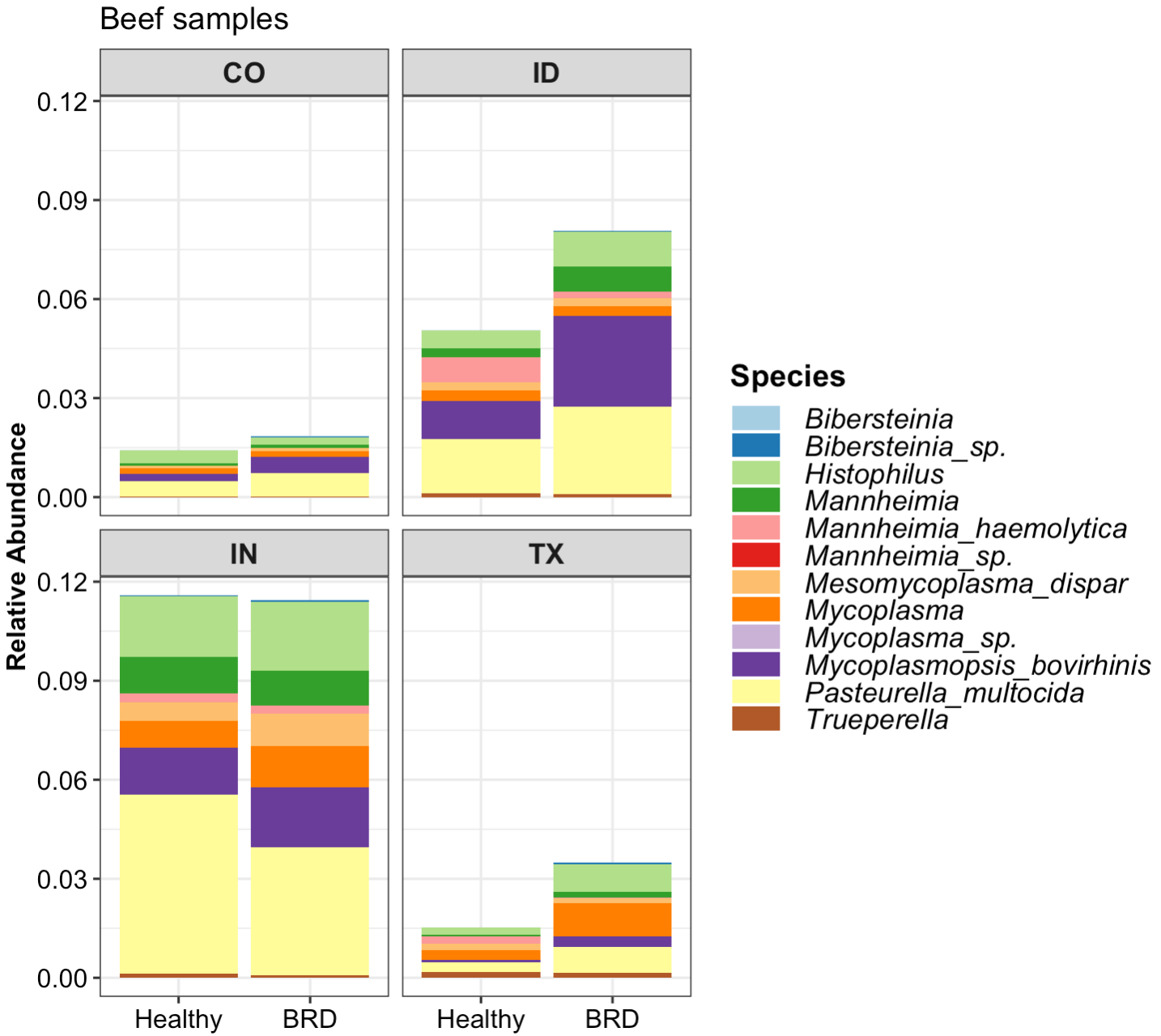


**Figure S1**. Relative abundance of BRD-pathobionts predicted by 16S rRNA gene sequencing in the beef cattle nasal microbiome.


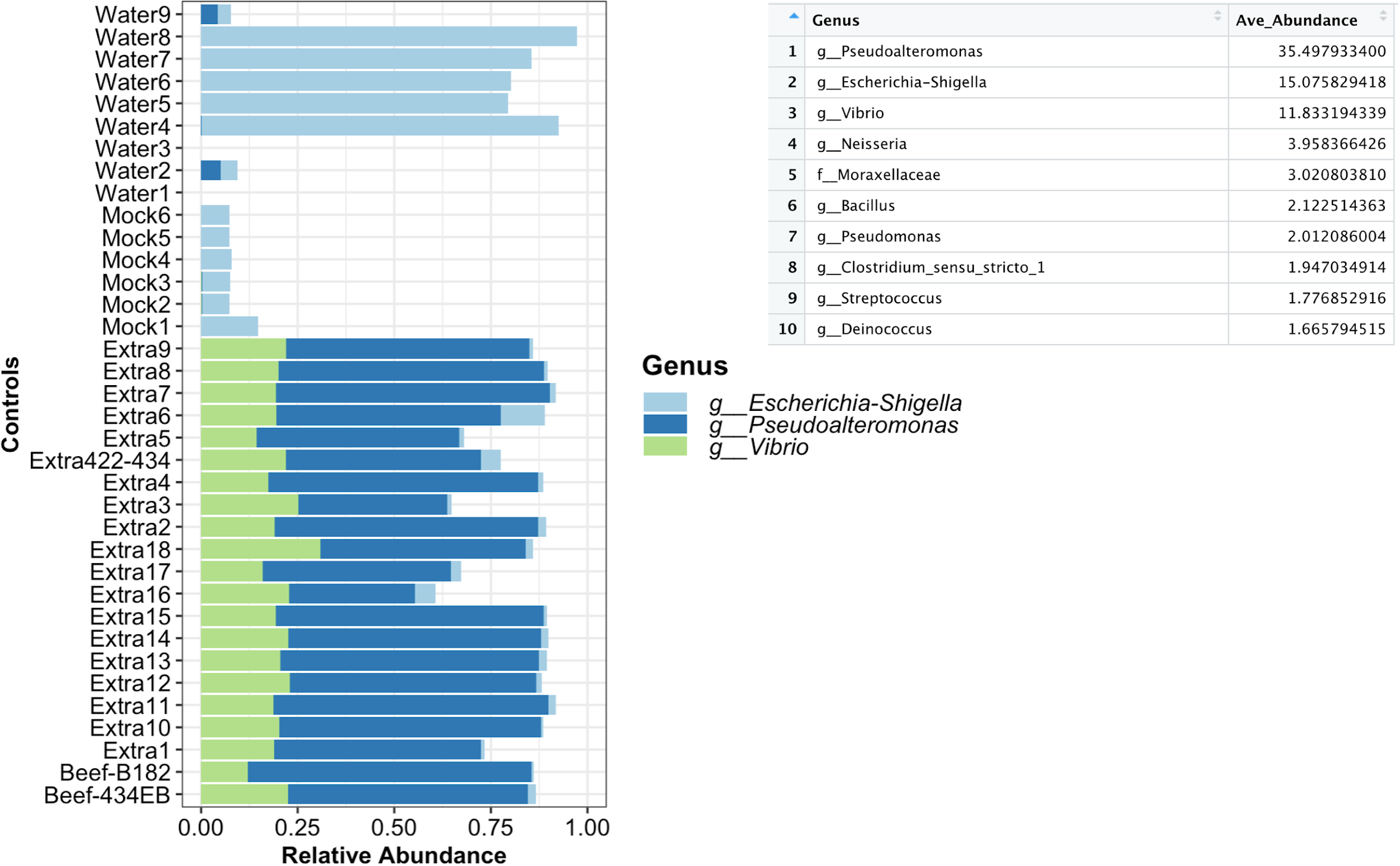


**Figure S2.** Bar plot showing relative abundance of the ASVs found in the controls including *Pseudoalteromonas* Controls included the water samples “water”, mock, and empty swabs (Beef and extra).


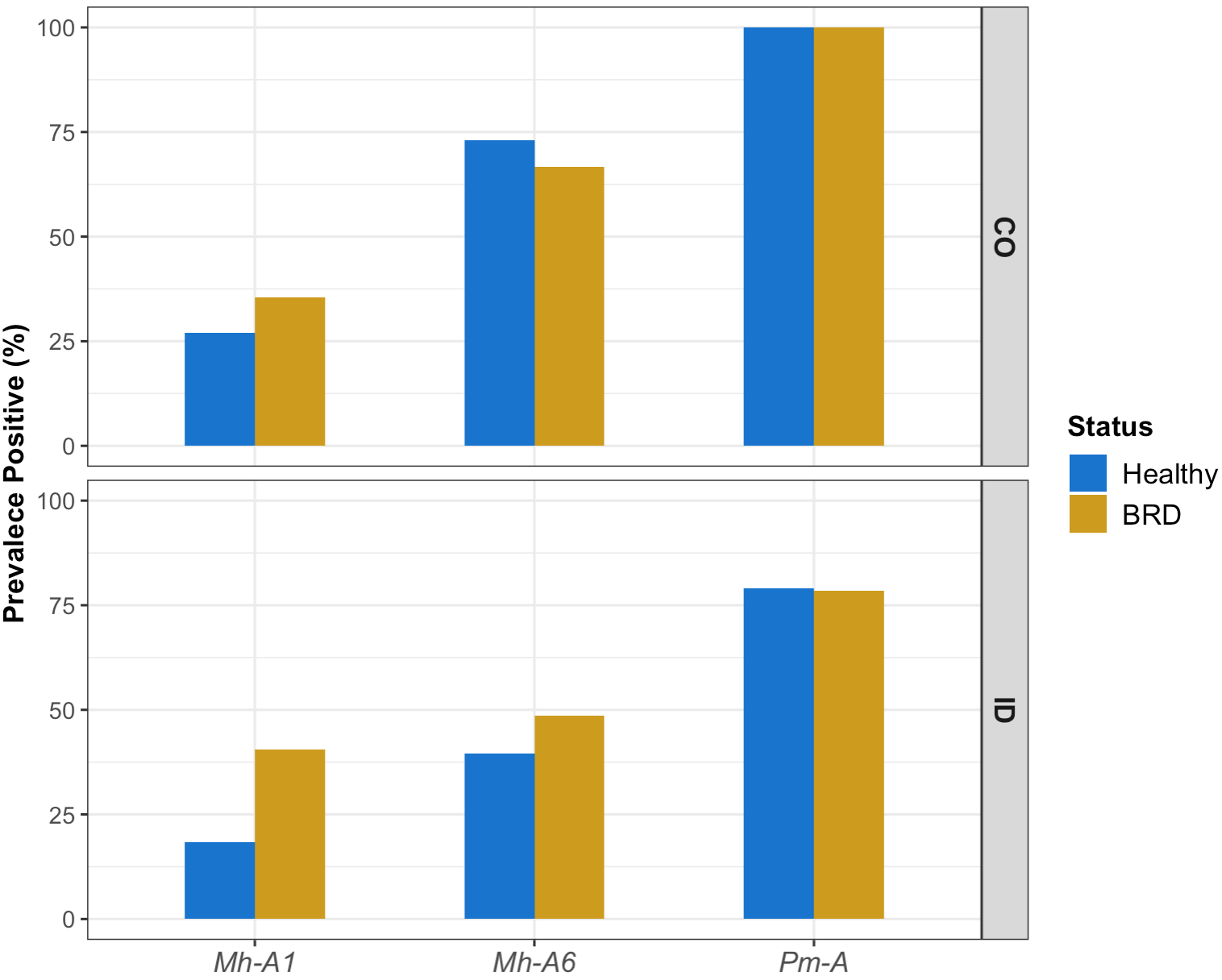


**Figure S3.** Prevalence of *M. haemolytica* serotype A1 and A6 and *P. multocida* serotype A in samples collected from beef cattle in CO and ID.

**BRD-pathobionts relative abundance**

Relative abundance for each of the BRD-pathobionts was calculated using the total bacterial abundance obtained via 16S rRNA qPCR. From the analysis, *H. somni* relative abundance in the CO and TX samples and *M. bovis* relative abundance in TX were significantly different between health-status groups (Figure S4). Furthermore, to ascertain the abundance of each pathobiont relative to the others within the same sample, a separate relative abundance was calculated by dividing the abundance of each pathobiont by the total abundance (sum) of the four pathogens in the sample. From the analysis, the relative abundance of *H. somni* and *P. multocida* compared to the total pathobiont abundance within the sample was significantly different in the IN and TX samples and dependent on disease status (Figure S5). The total bacterial abundance quantified via 16S rRNA qPCR was significantly different for all the four farms (Figure S6a). Samples from CO and IN had higher bacterial abundance than TX and ID. Lastly, no significant difference in total bacterial abundance between disease status groups was detected except for IN samples, where the BRD-affected group had higher bacterial abundance than the healthy group (Figure S6b).

**
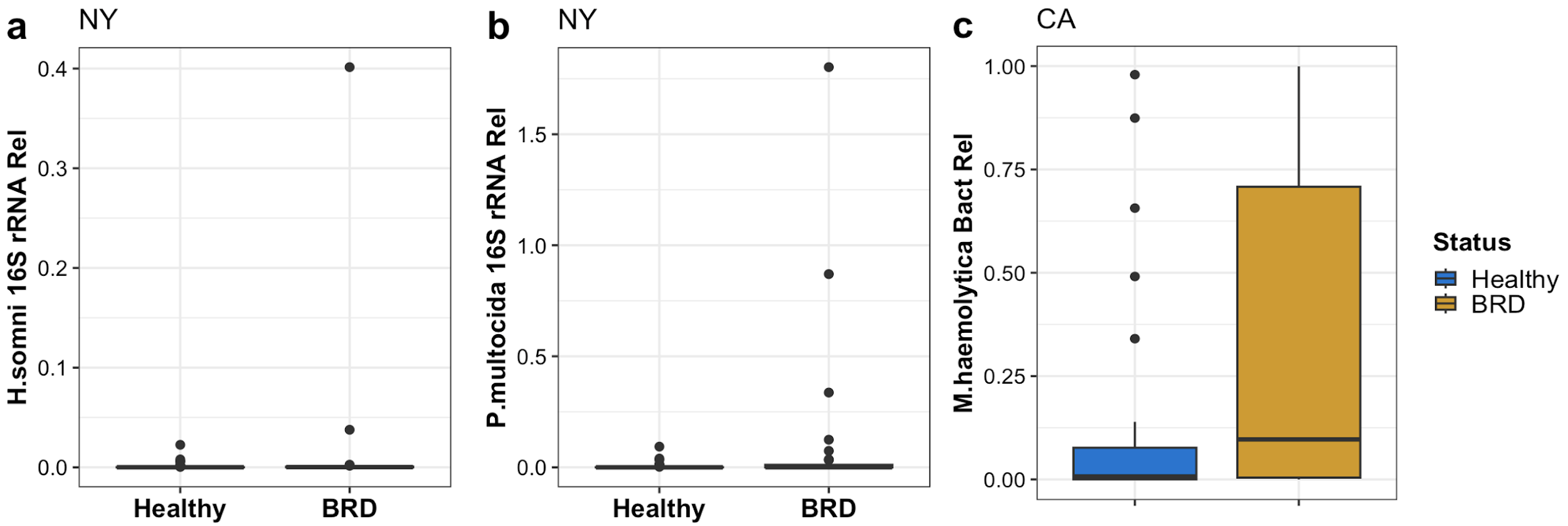
**

**Figure S4**. Relative abundance of *M. bovis* **(a)** and *P. multocida* **(b)** based on the 16S rRNA abundance and *M. haemolytica* relative abundance based on the four pathobionts **(c).**


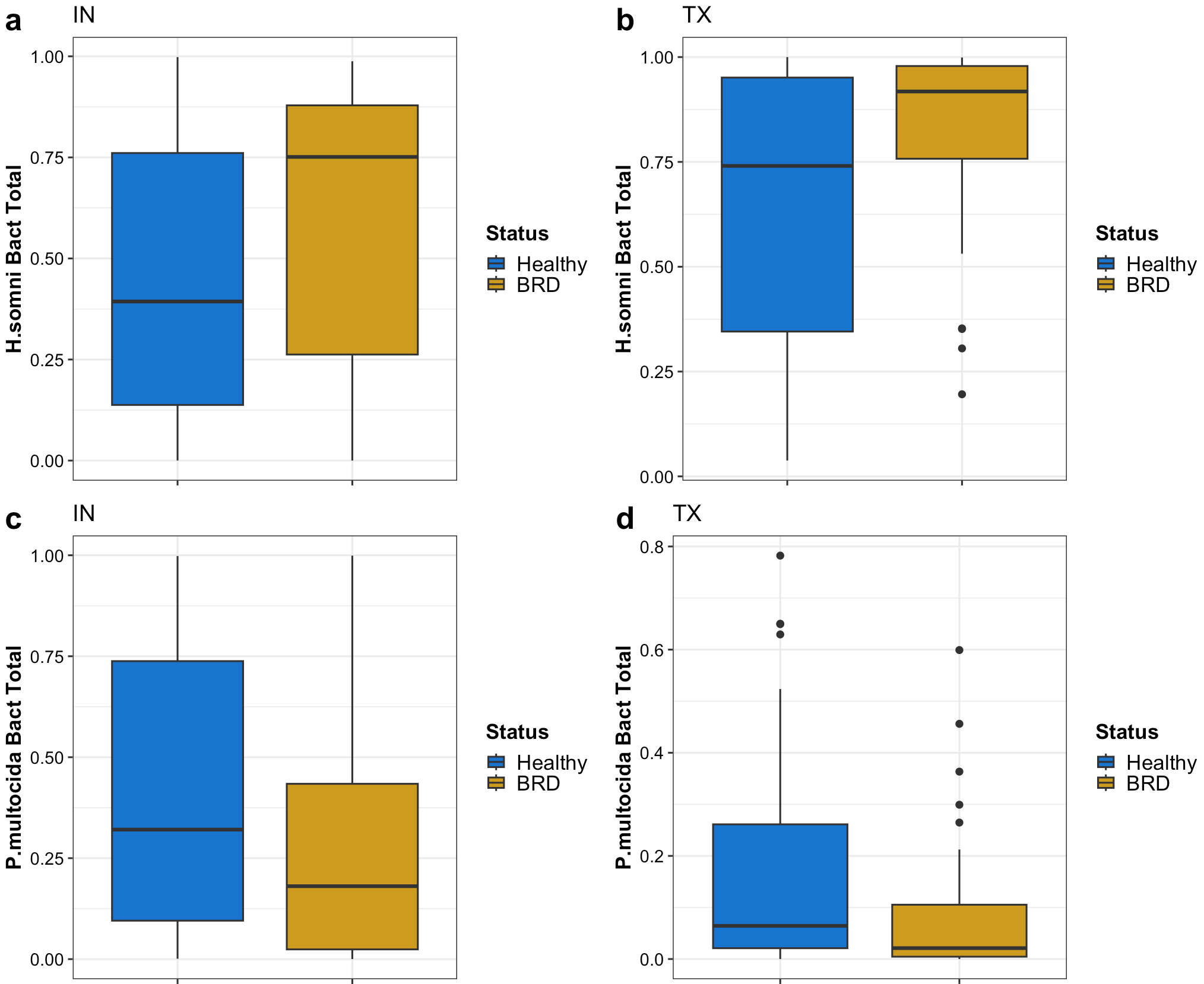


**Figure S5.** Relative abundance of *H.somni*  and *P. multocida* based on the four pathobionts from the samples collected in IN **(a,c)** and TX **(b,d).**

**
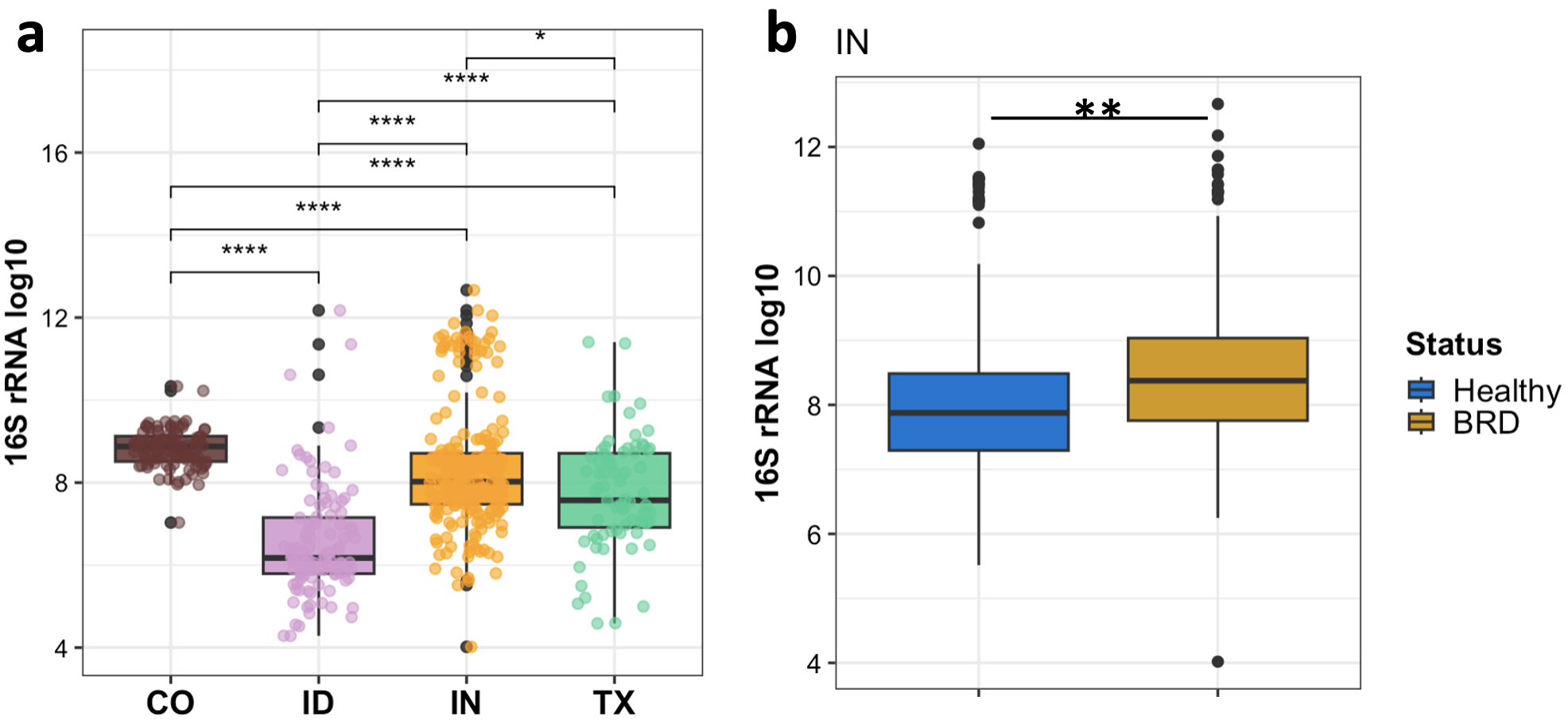
**

**Figure S6**. Total bacteria abundance quantified by 16S rRNA qPCR among the four farms (**a)** and between both disease statuses in IN **(b).**

**Comparison analysis between BRD-pathobiont prevalence and relative abundance between detected via 16S rRNA sequencing and qPCR**

In a comparative analysis, we examined the prevalence and relative abundance of ASVs identified as *Histophilus, P. multocida, M. haemolytica*, and *Mycoplas*ma in the 16S rRNA data, comparing it with the abundance and prevalence of BRD-pathobionts quantified via qPCR. The detection of *H. somni* based on qPCR was consistently above 75%, regardless of farm and disease status. However, *Histophilus* detection based on 16S rRNA sequencing was higher in the BRD-affected group, exceeding 50%, compared to their apparently healthy group independently of the farm (Figure S7). *M. haemolytica* detection based on qPCR was approximately below 75% in both groups, with a higher prevalence in the BRD-affected group. Nevertheless, using the 16S rRNA sequencing data, no ASVs were detected as *M. haemolytica*; hence, ASVs classified as *Mannhemia* genus were selected for the analysis. Interestingly, the detection of *Mannheimia* genera based on 16S rRNA exceeded 75% among all farms and disease statuses. Similar results were observed for *M. bovis*.

According to qPCR data, the prevalence of *M. bovis* was below 75% in all farms, with a higher prevalence in the BRD-affected group. Similarly, no *M. bovis* was detected in the 16S rRNA sequencing data; therefore, the genera *Mycoplasma* was selected instead. Once again, more *Mycoplasma* genera were detected by the sequencing data compared to the qPCR assays. Lastly, the prevalence of *P. multocida* was similar between qPCR and 16S rRNA sequencing, with an average prevalence based on farm and disease status ranging from 50% to 75% (Figure S7). Additionally, a correlation analysis aimed to explore the association between pathobiont relative abundance from 16S rRNA sequencing and qPCR, evaluating whether qPCR, with higher sensitivity, aligns with the sequencing-based relative abundance. Interestingly, only *M. bovis* relative abundance predicted by 16S rRNA and qPCR had a significant association (R=0.13, *p* = 0.0034), and no other significant associations were detected for *H. somni, P. multocida,* and *M. haemolytica.*

**
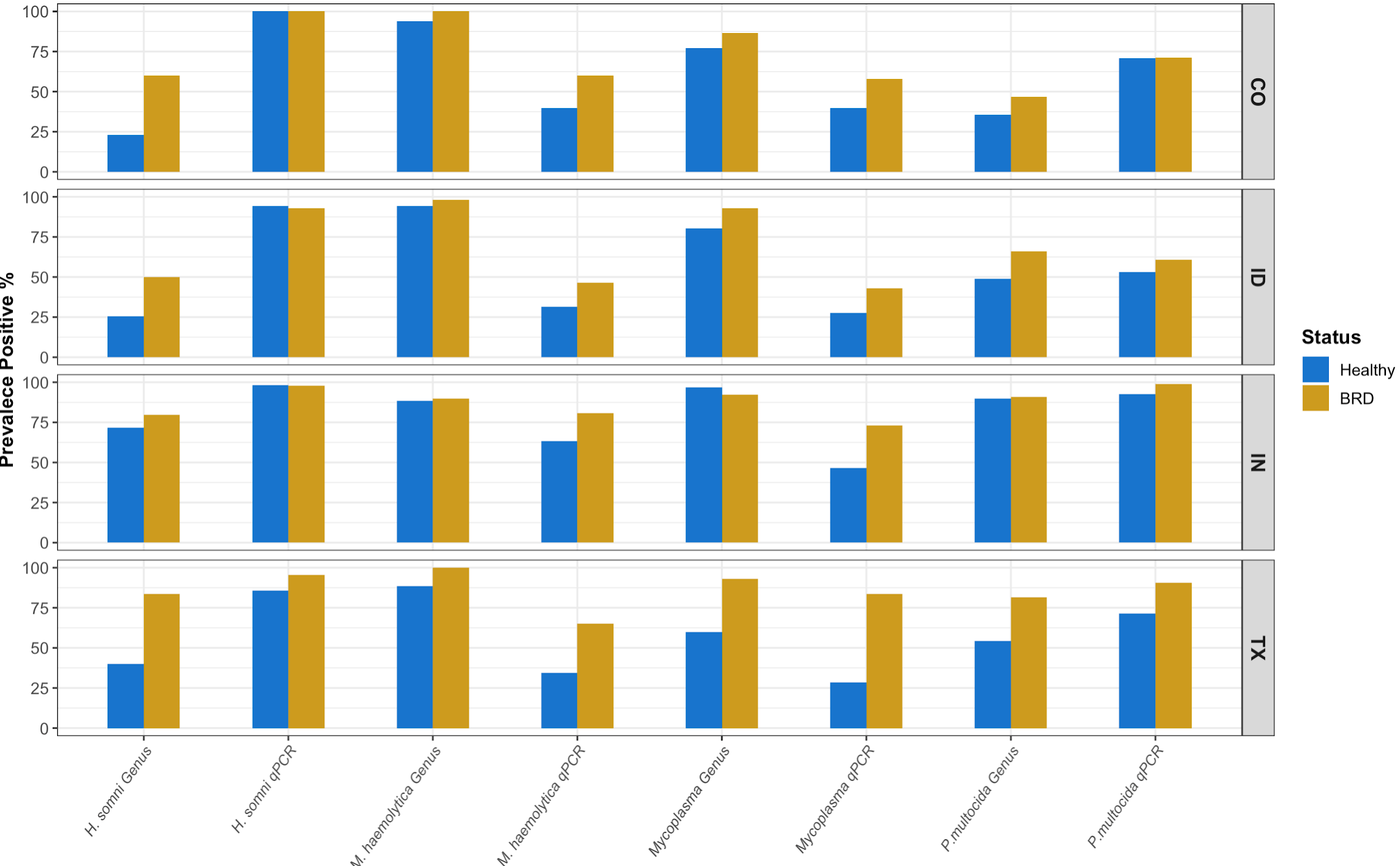
**

**Figure S7.** Prevalence of the BRD-pathobionts in the beef nasal cavity identified via 16S rRNA sequencing and quantified via qPCR.
